# Physical manoeuvers as a preventive intervention to manage vasovagal syncope: A systematic review

**DOI:** 10.1371/journal.pone.0212012

**Published:** 2019-02-28

**Authors:** Kim Dockx, Bert Avau, Emmy De Buck, Pascal Vranckx, Philippe Vandekerckhove

**Affiliations:** 1 Centre for Evidence-Based Practice (CEBaP), Belgian Red Cross, Mechelen, Belgium; 2 Cochrane Belgium, Belgian Centre for Evidence-Based Medicine (Cebam), Leuven, Belgium; 3 Faculty of Medicine, Department of Public Health and Primary Care, KU Leuven, Leuven, Belgium; 4 Department of Cardiology and Critical Care Medicine, Hartcentrum Hasselt, Jessa Ziekenhuis, Hasselt, Belgium; 5 Faculty of Medicine and Life Sciences, University of Hasselt, Hasselt, Belgium; 6 Belgian Red Cross, Mechelen, Belgium; Robert Gordon University, UNITED KINGDOM

## Abstract

**Aims:**

To summarize the best available evidence on the effectiveness of physical counterpressure manoeuvers (PCM) for vasovagal syncope management compared to a control intervention. Control interventions included either a PCM, no intervention, or other interventions feasible in a lay setting.

**Methods:**

A systematic literature search (March 21^st^ 2018) was performed in the Cochrane Central Register of Controlled Trials, MEDLINE, and Embase. PCM were subdivided into 1) PCM decreasing orthostatic load (PCMOL), 2) PCM shortening the hydrostatic column between heart and brain (PCMHC), 3) PCM using mechanical compression of the veins (PCMMC). The primary outcome was syncope, secondary outcomes included systolic blood pressure (SBP), diastolic blood pressure (DBP), mean arterial pressure (MAP), heart rate (HR), stroke volume (SV), cardiac output (CO), and total peripheral resistance (TPR). When possible, a random effects meta-analysis was performed. Odds ratios (OR) with 95% confidence intervals (CI) were calculated for dichotomous outcomes, and mean differences (MD) or standardized mean differences (SMD) were calculated for continuous outcomes. Heterogeneity was assessed by means of the I^2^ statistic. The total body of evidence was evaluated by means of the GRADE methodology.

**Results:**

Eleven trials involving 688 people with vasovagal syncope were included. Risk of bias was high in all included studies. The total body of evidence (GRADE) was considered to be low or very low. PCM were found to improve syncope as compared to control (OR: 0.52, 95% CI [0.33;0.81], p = 0.004). Similarly, before-and-after studies without a control group showed a significant reduction in syncope following PCM (OR: 0.01, 95%CI [0.00;0.01], p<0.001). No studies investigated PCMOL. PCMHC increased SBP, DBP, MAP, SV, and CO, and decreased HR. PCMMC increased SBP, DBP, and MAP.

**Conclusion:**

PCM may reduce syncope and increase SBP, DBP, and MAP. The effects on other outcomes are less clear. Additional high-quality studies are needed.

## Introduction

Syncope is defined as ‘a transient loss of consciousness due to transient global cerebral hypoperfusion characterized by rapid onset, short duration, and spontaneous complete recovery’[1]. It is a fairly common condition with an incidence rate of 6.2 per 1000 person-years, and becomes increasingly prevalent from the age of 70 years onwards[2]. Although the long-term prognosis of syncopal disorders is usually excellent, its impact on quality of life is substantial[3–5].

Three potential etiologies of syncope can be distinguished, namely reflex syncope (e.g. vasovagal syncope, situational syncope, and carotid sinus syndrome), orthostatic hypotension, and cardiac syncope[1]. Physical counterpressure manoeuvers (PCM) only relate to a subset of syncope events, such as vasovagal syncope and orthostatic hypotension as these are characterized by a prodromal phase (i.e. nausea, sweating, headache, pallor). Conventional vasovagal therapy aspires to prevent syncope recurrences and limit physical injuries by educating people on the benign nature of the condition, and underscoring the importance of avoiding potential triggers[6, 7]. In addition, an increased intake of dietary salt and fluid is recommended to augment the available blood volume[7–9]. More recently, PCM have been proposed as a valuable complement[7, 10, 11]. PCM aim to prevent peripheral pooling of blood by decreasing orthostatic load (e.g. sitting down), shortening the hydrostatic column between the heart and the brain (e.g. bending the head), and/or increasing the central blood flow by generating a mechanical compression of the veins (e.g. leg crossing)[7, 10, 12]. The use of PCM for syncope management shows great promise, however, the evidence has not yet been formally reviewed or synthesized.

Therefore, the objective of this systematic review was to summarize the best available evidence on the effectiveness of PCM for the management of vasovagal syncope. The primary goal was to determine the effect of PCM on syncope following prodromal symptoms. Secondary goals included examining the effects of PCM on hemodynamic parameters, comprising of systolic blood pressure (SBP), diastolic blood pressure (DBP), mean arterial pressure (MAP), heart rate (HR), stroke volume (SV), cardiac output (CO), and total peripheral resistance (TPR). Following PICO question was formulated to answer these research questions: In patients with vasovagal syncope (P), is the use of PCM during the prodromal phase (I), compared to not using PCM (C), safe and effective for the prevention of syncope (O)?

## Materials and methods

For this manuscript, we followed our previously published methodological charter[13], and made use of the reporting criteria provided in the PRISMA checklist (S1 File) [14].

### Eligibility criteria

#### Types of studies

All randomized controlled trials, quasi-randomized controlled trials, non-randomized controlled trials, before and after studies, and interrupted time series were considered for inclusion in the current review. Conference abstracts, cohort studies, case-control studies, case series, and cross-sectional studies were excluded. Only studies in English were included.

#### Types of participants

All subjects who presented with a history of vasovagal syncope were eligible for inclusion. Vasovagal syncope was defined as a neurally mediated syncope, whereby the cardiovascular reflexes become intermittently inappropriate in response to emotional or orthostatic stress, resulting in vasodilatation and/or bradycardia[15]. No restrictions were made with regards to gender, age, severity, or frequency of syncope occurrences. Subjects who presented with syncope in the context of a blood donation were excluded.

#### Types of interventions

Any study investigating the effectiveness of a PCM compared to a control intervention, or examining the pre to post effects of a PCM was included. A PCM was defined as any manoeuver, that is feasible to be performed in response to prodromal symptoms, in a lay setting, aimed to prevent peripheral pooling of blood and subsequent syncope by decreasing orthostatic load, shortening the hydrostatic column between the heart and the brain, and/or increasing the central blood flow by generating a mechanical compression of the veins[7, 10, 12]. All pharmacological, surgical, or other treatments that are not fit for a lay setting (e.g. breathing manoeuvers) were excluded. Control interventions included either a PCM, no intervention, or other interventions that are feasible in a lay setting (e.g. increasing dietary salt intake).

#### Types of outcome measures

The primary outcome was syncope. This was expressed as the number of people suffering from at least one syncope episode over a period of time.

Secondary outcomes included SBP, DBP, MAP, HR, SV, CO, and TPR. SBP (mmHg), DBP (mmHg), HR (bpm), and SV (%) were derived from the arterial pulse wave. MAP (mmHg) was calculated as the time integral over the beat-to-beat pressure recordings. CO (%) was defined as SV*HR. TPR (%) was calculated by dividing MAP by CO.

### Search methods

A systematic literature search was performed through electronic searches of the Cochrane Central Register of Controlled Trials (CENTRAL–the Cochrane Library), MEDLINE (PubMed interface), and Embase (embase.com interface). Tailored search strategies were developed for each of the databases. These can be found in S2 File. The search was performed on March 21^st^ 2018. Moreover, reference lists of all included trials and relevant systematic and narrative reviews, identified via the database searches, were hand searched.

### Data collection and quality assessment

Two reviewers independently screened 1) all search results based on title and abstract, 2) potentially relevant articles based on the full-text. Data of all included articles were extracted by a single reviewer onto a data collection form including citation details, trial setting, study population, intervention details, outcome measures, and results. Data extraction was double-checked by a second reviewer. Study authors were contacted for additional information when necessary.

The methodological quality of each of the included trials was independently assessed by two reviewers, according to the Cochrane risk of bias tool[16]. The following items were evaluated: sequence generation (randomization), allocation concealment, blinding of participants and personnel, blinding of outcome assessors, incomplete accounting of outcome events, selective outcome reporting, and other study limitations. Studies were determined to be at a low risk of bias if all items were assigned a low risk of bias, at an unclear risk of bias if one or more items were scored at an unclear risk of bias, and at a high risk of bias if one or more items were found to be at a high risk of bias. Any disagreements in study selection, data extraction, or quality assessment were resolved through discussion or, if necessary, through independent arbitration of a third reviewer.

The quality of the body of evidence was assessed for each of the outcomes of this review, using the methodology of the GRADE working group[17]. For each outcome, quality was assessed over 5 domains: limitations in study design (risk of bias), consistency, indirectness, imprecision of the results and publication bias. Evidence from experimental studies start at a high level of evidence. Evidence levels can range from high over moderate to low and very low.

### Data analysis

PCM were subdivided into three categories, namely 1) PCM decreasing orthostatic load, 2) PCM shortening the hydrostatic column between the heart and the brain, and 3) PCM generating a mechanical compression of the veins. The analyses were performed for each PCM category separately. In addition, an overall analysis, pooling data from all PCM, was performed for the primary outcome.

When possible, a random effects meta-analysis was performed. For dichotomous outcomes, the Maentel-Haenzel method was used, for continuous outcomes, the inverse variance method was used. In case of paired dichotomous outcomes (before-after measurements) the Becker-Balagtas method was applied[18, 19]. A conservative approach was applied for meta-analyses involving within-subject study designs, by ensuring that the number of participants in the before and after cohorts were well matched. This means that patients for whom no post-measurement was available, were excluded from the pre-post analysis[20]. Whenever a meta-analysis was not desirable, i.e. due to substantial differences between studies or when only one study was identified, a narrative description of the results was provided. Odds ratios (OR) with 95% confidence intervals (CI) were calculated for each of the dichotomous outcomes. Mean differences (MD) or standardized mean differences (SMD) were calculated for each of the continuous outcomes, as appropriate. Heterogeneity was assessed by means of the I^2^ statistic. When appropriate, a sensitivity analysis was performed including low risk of bias trials only. Cochrane’s Review Manager 5 software[21] was used for all analyses.

## Results

**Fig 1:**
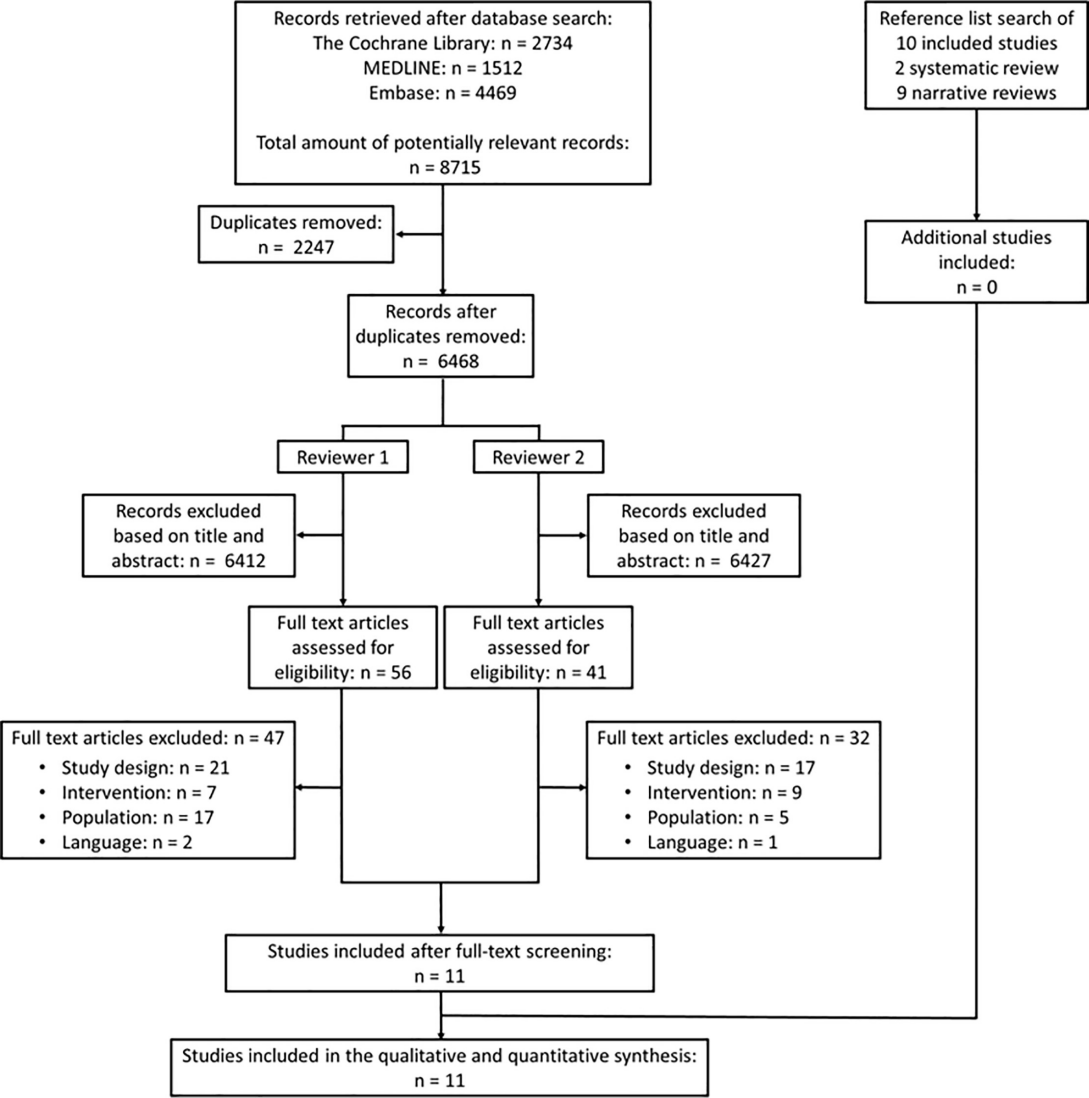
Study flow diagram.

A total of 8715 records were identified. Duplicates (n = 2247) were removed, resulting in 6468 titles and abstracts to be reviewed. Of these, 56 full-text articles were assessed for eligibility by reviewer 1 and 41 were assessed by reviewer 2. The majority were excluded because they did not adhere to the predefined inclusion criteria in terms of design (n = 23), intervention (n = 14), population (n = 22), or language (n = 2) (S1 Table). Eleven studies were found eligible for inclusion[22–32], and were included in the qualitative and quantitative synthesis. A study flow diagram can be found in (Fig 1).

### Study characteristics

A detailed overview of the study characteristics can be found in S2 Table. All included studies had an experimental design, with the vast majority being within-subject studies (n = 8). There were only two randomized controlled trials (RCT), and one non-RCT. A total of 688 people with vasovagal syncope were included. These involved 293 males, 369 females, and 26 participants for whom gender was not reported. Just over half of the trials (54.5%) involved fewer than 50 participants, four studies included 50–100 participants, and only one study recruited over 100 participants.

A wide variety of PCM were examined. No trial examined the effects of PCM decreasing orthostatic load, one trial looked at PCM shortening the hydrostatic column between the heart and the brain, i.e. bending the head, and 11 trials studied PCM generating mechanical compression of the veins, including hand grip (n = 1), leg crossing (n = 1), squat (n = 1), lower body muscle tension (n = 2), whole body muscle tensing (n = 1), and leg crossing with muscle tension (n = 3).

The effectiveness of PCM was assessed either in a daily life setting or in a laboratory setting. A daily life intervention was applied in 7 trials, for which the follow-up period ranged between 2 to 24 months. Assessments in the laboratory setting included tilt-testing or standing-up protocols. Tilt testing procedures were designed to trigger prodromal syncope symptoms. If no symptoms occurred, nitroglycerine was administered in five trials. Standing-up protocols included the use of a lying-to-standing test to induce hypotension.

### Risk of bias

**Fig 2:**
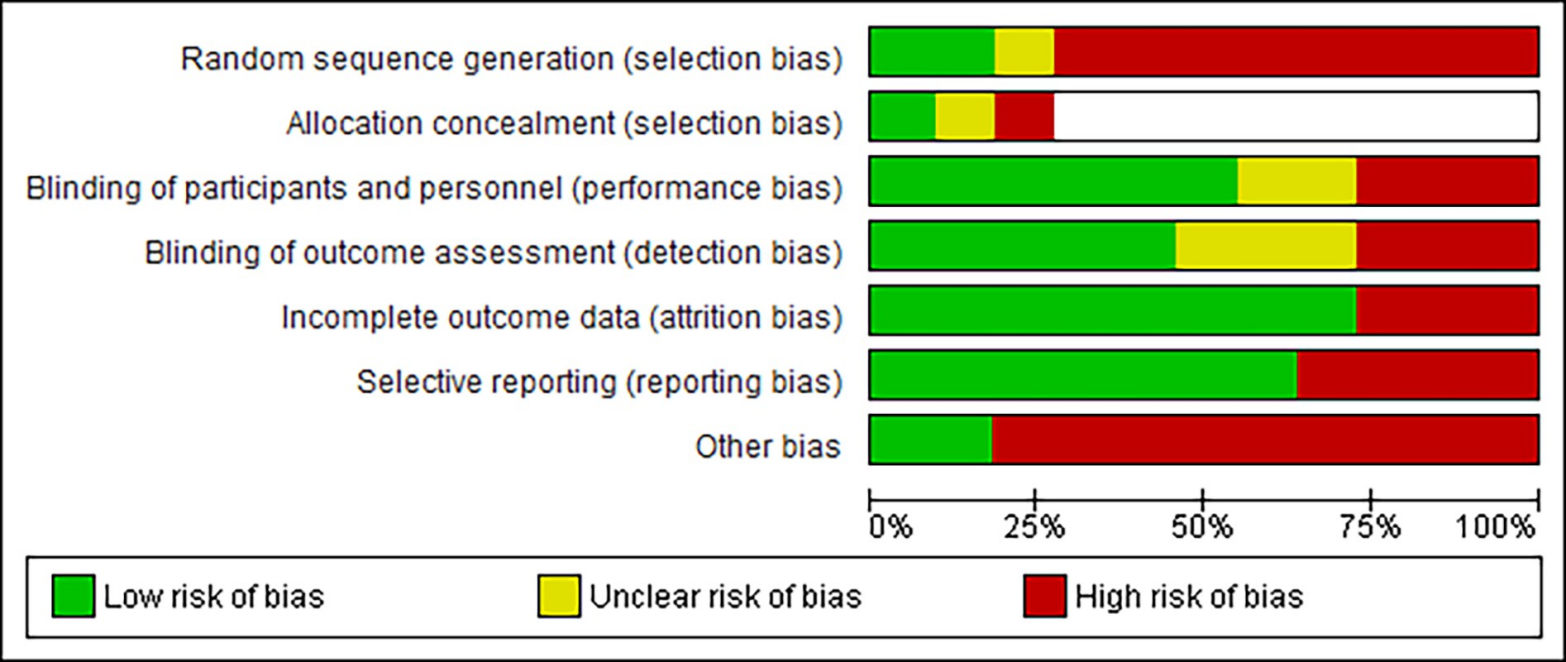
Risk of bias graph: Review authors’ judgments about each risk of bias item presented as percentage across all included studies. (White = not applicable).

**Fig 3:**
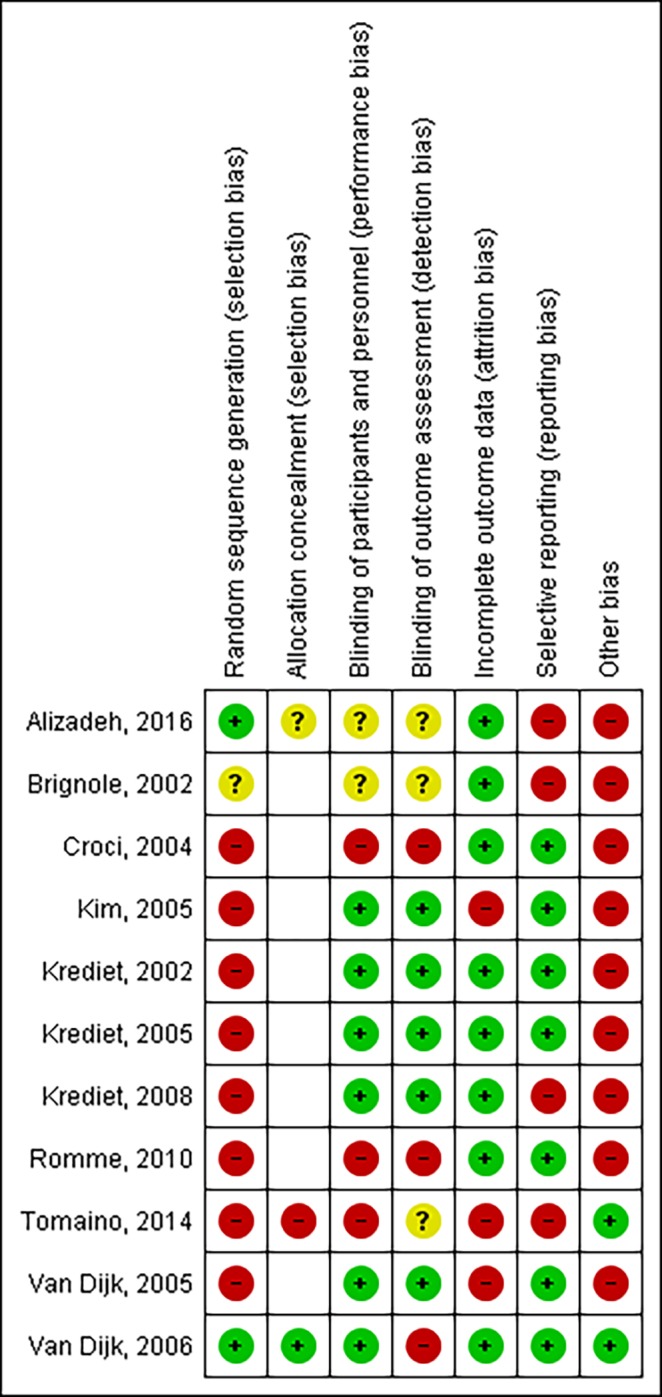
Risk of bias summary: Review authors’ judgment about each risk of bias item for each included study. (White = not applicable).

An overview of the methodological quality is reported in (Figs 2 and 3). All studies were found to be at a high risk of bias. In 73% of all trials, randomization was not performed. As such, in most trials (n = 8) allocation concealment was considered to be irrelevant. Blinding of 1) participants and personnel, and 2) outcome assessment was deemed to be at a low risk of bias in 55% and 45% of studies, respectively. Mostly, this low risk of bias was assigned because of the use of objective outcome measures which would not have been affected by blinding. Incomplete outcome reporting was considered to be high in 27% of trials, as these had suffered a considerable loss to follow-up. A high risk of bias for selective outcome reporting was found in 36% of trials, as certain data were not well reported and could therefore not be extracted. Finally, 82% had a high risk for other biases, because they did not have a control group or did not report baseline scores.

### Level of evidence of the primary outcome: Syncope

The level of evidence of the overall body of evidence is low or very low. A detailed overview can be found in S3 Table. A detailed overview of the effect of PCM on syncope can be found in S4 Table.

#### Daily life interventions

**Fig 4:**
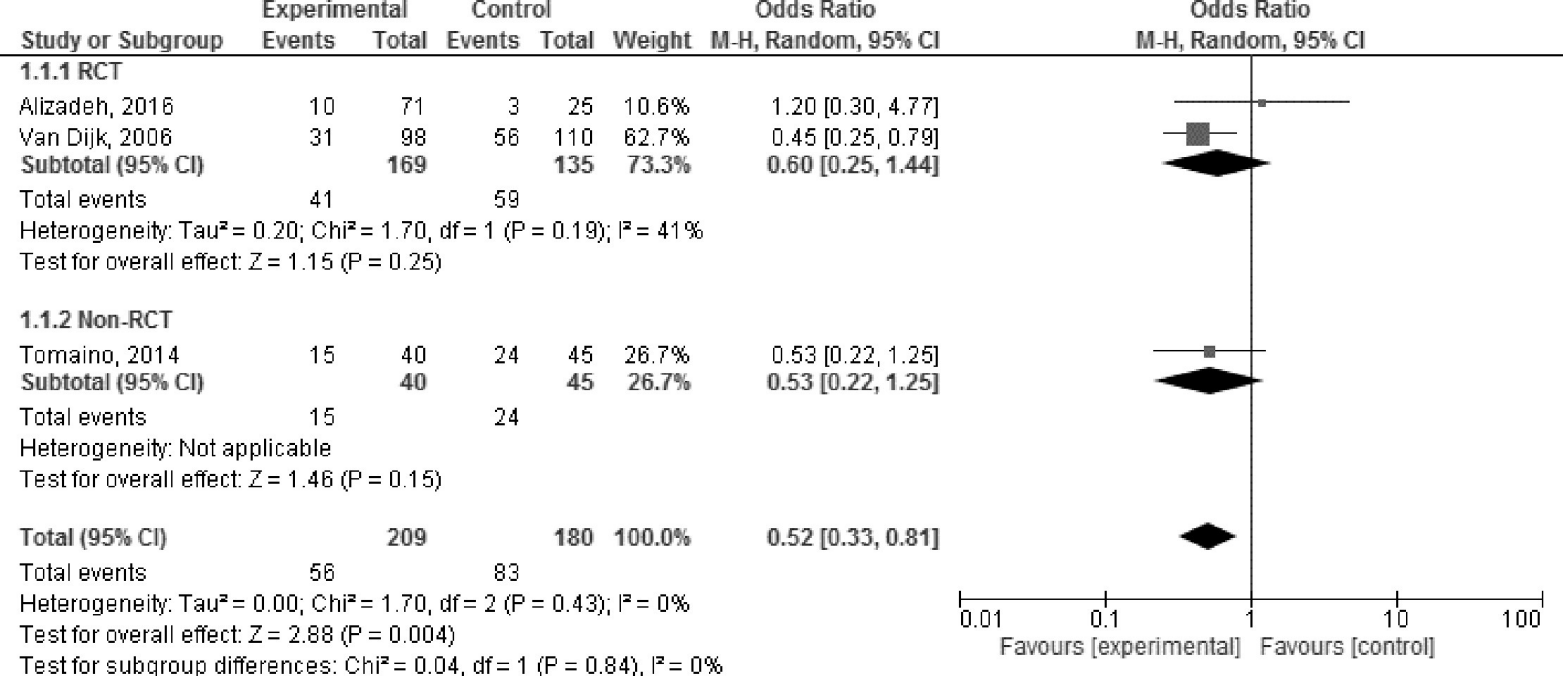
Meta-analysis on the impact of physical manoeuvers on syncope prevalence, as compared to a control intervention–daily life setting.

Three trials examined the impact of PCM on syncope as compared to a control group. These trials examined a set of PCM, involving hand grip, squat, leg crossing, arm tensing, or lower body muscle tensing. A meta-analysis indicated a significant difference between the intervention cohort which used PCM to avoid a syncopal event, and control interventions (Fig 4). OR: 0.52, 95% CI [0.33;0.81], p = 0.004, I^2^ = 0% [0.0%; 87.8%])(22, 30, 32). A sub-analysis including only RCT trials[22, 30] showed a trend towards significance (Fig 4). OR: 0.60, 95% CI [0.25;1.44], p = 0.25, I^2^ = 41%).

**Fig 5:**
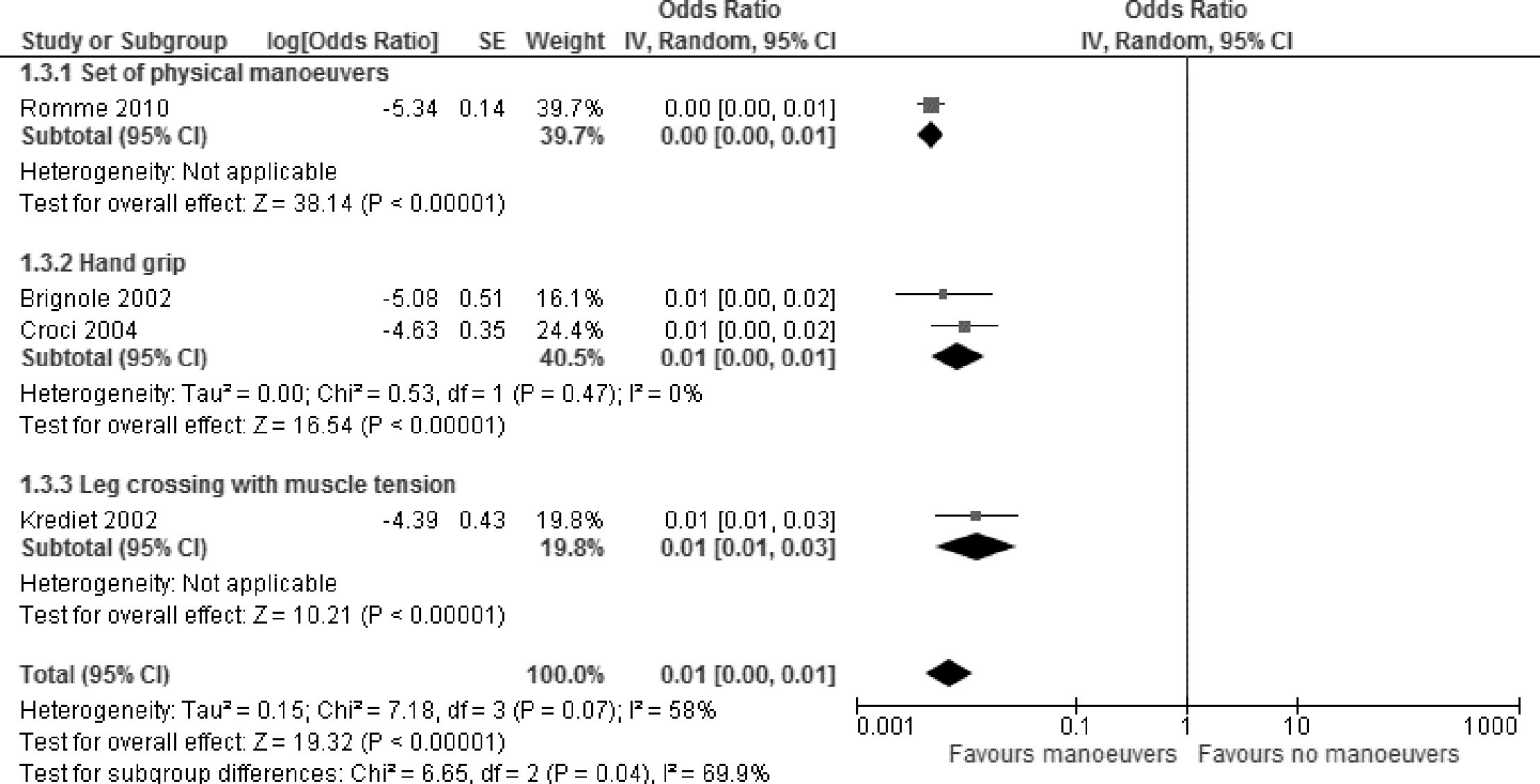
Meta-analysis on the impact of physical manoeuvers on syncope prevalence in before-and-after study designs–daily life setting.

Four studies investigated the effectiveness of PCM by comparing the number of syncopal events before and after intervention, without the inclusion of a control cohort. A meta-analysis of these four before-and-after studies showed a significant reduction of syncope following PCM (Fig 5). OR: 0.01, 95% CI [0.00;0.01], p<0.001, I^2^ = 58% [0.0%; 86.1%])[23, 24, 27, 28]. Substantial heterogeneity was found, which might be explained by the different types of PCM that were applied in the different studies, i.e. arm tensing, hand grip, leg crossing, lower body muscle tensing, leg crossing with lower body muscle tensing, and squat. An explorative subgroup analysis, stratifying the effect by type of PCM could possibly explain the observed heterogeneity (Fig 5). Test for subgroup differences: p = 0.04, I^2^ = 69%).

#### Laboratory trials

**Fig 6:**
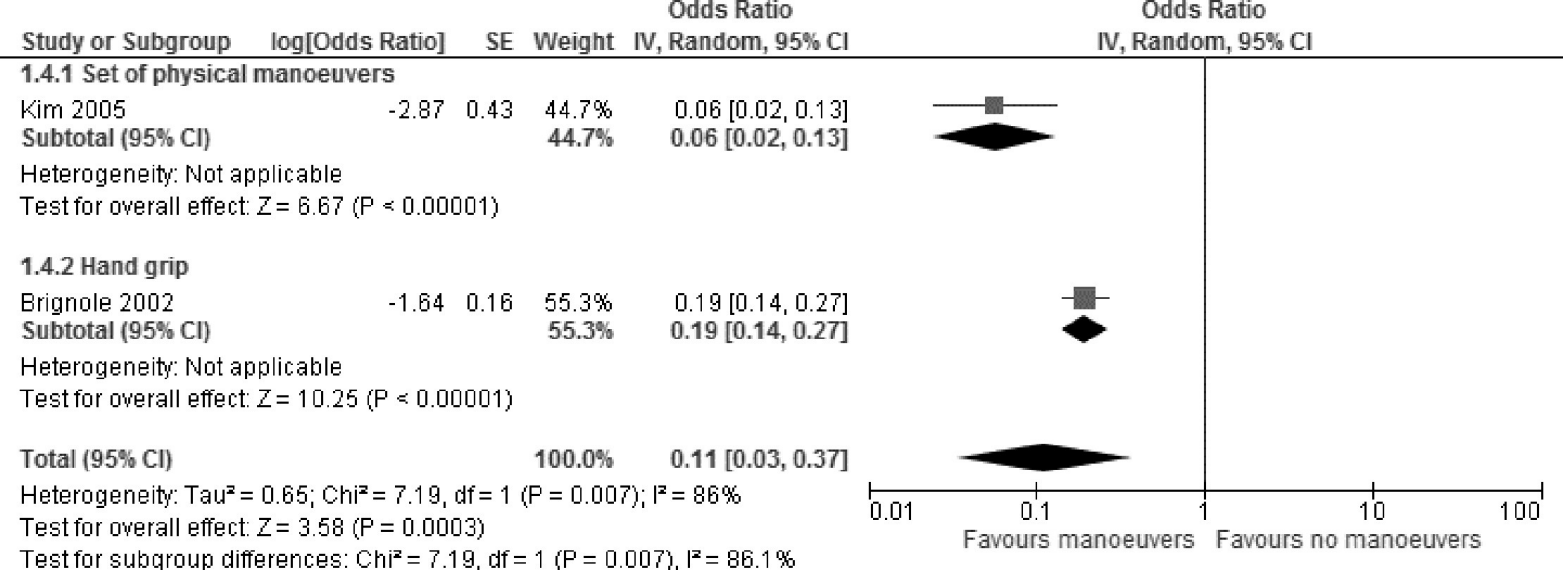
Meta-analysis on the impact of physical manoeuvers on syncope prevalence, compared to a control intervention–laboratory setting.

Two laboratory trials examined the impact of PCM on syncope as compared to a control group. A meta-analysis showed a significant reduction of syncope following PCM (Fig 6). OR: 0.11, 95% CI [0.03;0.37], p = 0.007, I^2^ = 86%)[23, 26]. The fact that the two studies used different PCM (i.e. hand grip, leg crossing with muscle tension, and squat) might be an explanation for the observed heterogeneity.

A before-and-after study demonstrated a significant reduction of syncope from pre to post intervention following PCM (combined RR: 0.63, 95% CI [0.39;0.97], p = 0.07)[31]. The PCM in this study included hand grip, leg crossing with muscle tension, and squat.

### Level of evidence of the secondary outcomes

Secondary outcomes, i.e. SBP, DBP, MAP, HR, SV, CO, and TPR, were all measured during laboratory trials. None of the daily life interventions incorporated any of the secondary outcomes. The level of evidence of the overall body of evidence is low or very low for all outcomes. A detailed overview of the quality of the evidence can be found in S3 Table, while an overview of the findings can be found in S4 Table.

#### PCM decreasing orthostatic load

No trial on PCM decreasing orthostatic load was identified.

#### PCM shortening the hydrostatic column

One trial studied the effect of PCM shortening the hydrostatic column between the heart and the brain, by examining the effect of bending the head between the knees^,^[25]. The study by Krediet et al. demonstrated a significant increase in SBP, DBP, MAP, SV, and CO, and a significant decrease in HR following head bent between the knees. A significant effect on TPR could not be demonstrated.

#### PCM using mechanical compression of the veins: Hand grip

One trial examined the effect of hand grip as a PCM[23]. Hand grip was shown to significantly increase SBP and DBP as compared to a control intervention. A significant effect on HR could not be found. There are currently no studies available investigating the effects of hand grip on MAP, SV, CO, or TPR.

#### PCM using mechanical compression of the veins: Leg crossing

One trial examined the effects of leg crossing[29]. SBP, DBP, MAP, SV, and CO were shown to increase significantly following leg crossing. An effect of leg crossing on HR could not be demonstrated. TPR was shown to reduce.

#### PCM using mechanical compression of the veins: Squatting

One study investigated the impact of squat[25]. It showed an increase in SBP, DBP, MAP, SV, and CO following squat. HR and TPR did not significantly change following squat.

#### PCM using mechanical compression of the veins: Lower body muscle tension

Two trials investigated the effects of lower body muscle tension[25, 26]. One trial showed an increased SBP, DBP, MAP, and CO as compared to control. An effect of lower body muscle tension on TPR could not be demonstrated.

In addition, a before-and-after study showed SBP, DBP, MAP, HR, SV, and CO significantly increased from pre to post intervention[25]. An effect on TPR could not be shown[25]. These effects proved to be inferior to leg crossing with muscle tension in terms of SBP, MAP, SV, and CO, but no significant difference could be demonstrated in terms of DBP, HR and TPR[25].

#### PCM using mechanical compression of the veins: Whole body muscle tension

One study examined the impact of whole body muscle tension[25]. This study showed a significant increase in SBP, DBP, MAP, HR, SV, and CO from pre to post intervention. An effect of whole body muscle tension on TPR could not be demonstrated. The impact was found to be inferior to leg crossing with muscle tension in terms of SBP and MAP, while a difference in the effects on DBP, HR, SV, CO, and TPR could not be demonstrated.

#### PCM using mechanical compression of the veins: Leg crossing with muscle tension

Three trials investigated the effects of leg crossing with muscle tension[25, 27, 29]. All trials found a significant increase in SBP, DBP, MAP, HR, SV, and CO. TPR significantly decreased in one study[29], while another trial could not demonstrate a significant effect[25].

The impact of leg crossing with muscle tension proved to be superior to lower body muscle tension in terms of SBP, DBP, MAP, SV, and CO, but similar for HR and TPR. The effect of leg crossing with muscle tension was superior to whole body muscle tension in terms of SBP and MAP, but similar regarding DBP, HR, SV, CO and TPR[25].

## Discussion

The aim of this systematic review was to explore whether PCM are effective for the management of vasovagal syncope. Although PCM are already widely encouraged in clinical practice, the evidence on its effectiveness was limited and had never been formally reviewed. Based on the current findings, PCM may reduce syncope, and increase SBP, DBP, and MAP. However, the impact on other outcomes such as HR, SV, CO, and TPR is less clear. In addition, it is yet to be fully established which type of PCM is most beneficial and feasible for use in specific clinical populations.

The evidence presented in this systematic review largely corresponds to earlier work which has suggested that PCM are effective, easy to apply, and inexpensive methods for handling a syncope episode[7, 10, 33]. In a meta-analysis, including 389 people with vasovagal syncope, we showed that the odds of developing a syncope episode in those who use PCM are approximately half (OR: 0.52) the odds of controls who are not using PCM. Although this is based on low quality evidence, these findings do advocate the use of PCM as a preventive intervention and support the continued integration of PCM into syncope therapy.

In most of these studies, participants were free to choose which PCM they wished to apply in case of an emerging syncope episode. As such, a wide variety of PCM were used, making it unclear which type of PCM is most useful for lowering syncope. There was only one study available which compared the effects of two types of PCM on syncope, namely squat versus hand grip, but this study could not demonstrate a significant difference between both interventions[22]. Further study is warranted to compare the effectiveness and feasibility of various PCM. Particularly in older or diseased populations, certain PCM, i.e. squat or leg crossing, may prove to be inappropriate due to a great challenge of balance and coordination[34]. Possibly, PCM reducing orthostatic load, such as sitting down, might be more convenient and equally successful[35].

The underlying mechanisms of PCM are presently unclear. PCM are thought to reduce syncope by either decreasing orthostatic load, increasing central blood flow, and/or shortening the hydrostatic column between heart and brain[7, 10, 12]. However, their impact on hemodynamic parameters such as SBP, DBP, MAP, HR, SV, CO, and TPR remains speculative and is merely based on case reports and small uncontrolled trials. Based on the findings from the current systematic review, low to very low quality evidence suggests that PCM are beneficial for increasing SBP, DBP, and MAP. Although meta-analyses could not be performed due to a lack of data and large heterogeneity between trials, the effects on blood pressure are consistent throughout all trials. Evidence on the impact of PCM on HR, SV, CO and TPR varied, depending on study and maneuver tested. Definite conclusions were hampered due to imprecise outcomes, as a result of a lack of data. This is in contrast to earlier narrative reviews, which pointed out that PCM likely result in an increase of SV, CO and systemic vascular resistance[7, 10, 33]. Whether or not the effects of PCM reach a minimal important difference is hard to say. PCM may shift blood pressure levels from just below to just above the critical threshold, thus resulting in a clinically important effect[33].

This review knows several strengths. We searched for relevant studies in manifold research databases up to March 21^st^ 2018. All aspects of the review process were performed in duplicate. The study quality of each included trial was thoroughly examined using the Cochrane risk of bias tool[16]. In addition, GRADE methodology was used to judge the overall quality of the evidence.

There are limitations to consider as well. First, we managed to include only a limited number of studies involving a limited number of patients, all with a high risk of bias. Since several trials were performed by the same research group, there might also be a potential overlap of patients. Due to the small number of studies available, and a large heterogeneity between trials regarding to the PCM and outcome measures used, only few meta-analyses could be performed. Those meta-analyses that were performed included only two to four trials. In addition, most studies are not recently published, which increases the odds for publication bias [36]. Such publication bias could not be formally tested as the meta-analyses performed involved only a limited number of studies. Furthermore, small meta-analyses could lead to a false assumption of homogeneity, and limit the generalizability of the results.

## Conclusions

In conclusion, we found low- to very low-quality evidence suggesting that PCM are a useful preventive method for reducing syncope, and increasing SBP, DBP, and MAP. Additional high-quality studies are needed to substantiate these findings, and to determine which type of PCM is most beneficial.

## Supporting information

S1 File PRISMA checklist. (PDF)

S2 File Search strategy. (PDF)

S1 Table Characteristics of excluded studies. (PDF)

S2 Table Characteristics of included studies. (PDF)

S3 Table Quality of the evidence (GRADE). (PDF)

S4 Table Synthesis of findings. (PDF)
