# Supplementary material for: Physical manoeuvers as a preventive intervention to manage vasovagal syncope: A systematic review
Source: PLoS One. 2019 Feb 28;14(2):e0212012. doi: 10.1371/journal.pone.0212012 (PMC6395036; doi:10.1371/journal.pone.0212012)
Supplement: S2 File — (PDF) [file pone.0212012.s002.pdf]

The Cochrane Library:

1. [mh "syncope"] OR faint*:ti,ab,kw OR syncope*:ti,ab,kw OR (drop NEXT/1 attack*):ti,ab,kw OR vasovagal:ti,ab,kw OR lipothymi*:ti,ab,kw OR [mh "hypotension"] OR hypotens*:ti,ab,kw OR [mh "tilt-table test"] OR (orthostatic NEXT/1 tolerance):ti,ab,kw
2. [mh "posture"] OR posture:ti,ab,kw OR (leg:ti,ab,kw AND cross*:ti,ab,kw) (legs:ti,ab,kw AND cross*:ti,ab,kw) OR (muscle NEXT/1 tens*):ti,ab,kw OR arm:ti,ab,kw OR squat*:ti,ab,kw OR stand*:ti,ab,kw OR (leg:ti,ab,kw AND rais*:ti,ab,kw) OR (legs:ti,ab,kw AND rais*:ti,ab,kw) OR [mh "head-down tilt"] OR trendelenburg:ti,ab,kw OR (lie NEXT/1 down):ti,ab,kw OR (lying NEXT/1 down):ti,ab,kw
3. 1 AND 2

MEDLINE (via PubMed interface):

1. "syncope"[MeSH] OR faint*[TIAB] OR syncop*[TIAB] OR drop attack*[TIAB] OR vasovagal[TIAB] OR lipothymi*[TIAB] OR "hypotension"[MeSH] OR hypotens*[TIAB] OR "tilt-table test"[MeSH] OR "orthostatic tolerance"[TIAB] OR "orthostatic intolerance"[MeSH] OR "orthostatic intolerance"[TIAB]
2. "Primary Prevention"[Mesh:NoExp] OR "Health Education"[Mesh] OR "Health Behavior"[Mesh:NoExp] OR "Preventive Medicine"[Mesh] OR "Prevention and control "[Subheading] OR prevent*[TIAB]
3. "Posture"[Mesh] OR posture[TIAB] OR (leg[TIAB] AND cross*[TIAB]) OR (legs[TIAB] AND cross*[TIAB]) OR muscle tens*[TIAB] OR arm[TIAB] OR squat*[TIAB] OR stand*[TIAB] OR (leg[TIAB] AND rais*[TIAB]) OR (legs[TIAB] AND rais*[TIAB]) OR "head-down tilt"[MeSH] OR trendelenburg[TIAB] OR lie down[TIAB] OR lying down[TIAB]
4. 1-3 AND

Embase (via Embase.com interface):

1. 'faintness'/exp OR fain*:ab,ti OR syncop*:ab,ti OR (drop NEXT/1 attack*):ab,ti OR vasovagal:ab,ti OR lipothymi*:ab,ti OR 'hypotension'/exp OR hypotens*:ab,ti OR 'tilt table test'/exp OR (orthostatic NEXT/1 tolerance):ab,ti OR 'orthostatic intolerance'/exp OR (orthostatic NEXT/1 intolerance):ab,ti
2. 'prevention'/exp OR 'health education'/exp OR 'health behavior'/exp OR 'self examination'/de OR 'preventive medicine'/exp OR *'prevention':lnk OR* 'risk factor'/exp OR prevent*:ab,ti
3. 'body posture'/exp OR posture:ab,ti OR (leg:ab,ti AND cross*:ab,ti) OR (legs:ab,ti AND cross*:ab,ti) OR (muscle NEXT/1 tens*):ab,ti OR arm:ab,ti OR squat*:ab,ti OR stand*:ab,ti OR (leg:ab,ti AND rais*:ab,ti) OR (legs:ab,ti AND rais*:ab,ti) OR (head down NEXT/1 tilt*):ab,ti OR trendelenburg:ab,ti OR (lie NEXT/1 down):ab,ti OR (lying NEXT/1 down):ab,ti
4. 1-3 AND
