# Supplementary material for: Physical manoeuvers as a preventive intervention to manage vasovagal syncope: A systematic review
Source: PLoS One. 2019 Feb 28;14(2):e0212012. doi: 10.1371/journal.pone.0212012 (PMC6395036; doi:10.1371/journal.pone.0212012)
Supplement: S1 Table — (PDF) [file pone.0212012.s003.pdf]

Study	Reason for exclusion
SPRINT trial, 2016	Design – conference abstract
Arnold, 2013	Design – narrative review
Backon, 1991	Population – blood donors
Bejiqi, 2016	Design – conference abstract
Berry, 2006	Intervention – physical manoeuver not as preventive measure
Blanc, 2010	Design – editorial
Bouvette, 1996	Population – orthostatic hypotension
Clarke, 2010	Population – orthostatic hypotension
Cooper, 2011	Design – guideline
De Bruine, 2016	Intervention – physical manoeuver not in response to prodromal symptoms
Denq, 1997	Intervention – no physical manoeuver
Ditto, 2003	Population – blood donors
Ditto, 2009	Population – blood donors
Ditto, 2010	Population – blood donors
Ditto, 2013	Population – blood donors
Eschlböck, 2017	Design – systematic review
Figueroa, 2015	Intervention – no physical manoeuver
Foulds, 1990	Intervention – no syncope trigger
France, 2006	Population – healthy subjects
France, 2010	Population – blood donors
Gorelik, 2004	Intervention – no physical manoeuver
Goswami, 2017	Design – narrative review
Groothuis, 2007	Population – healthy subjects
Harms, 2010	Population – Orthostatic hypotension
Hasegawa, 2000	Intervention – no physical manoeuver
Hockin, 2017	Design – conference abstract
Inamura, 1995	Intervention – no syncope trigger
Joseph, 2017	Design – narrative review
Kozak, 1981	Design – case study
Krediet, 2005	Design – case study
Krediet, 2006	Population – healthy subjects
Krediet, 2007	Population – Orthostatic hypotension
Kweon, 2012	Population – coronary bypass patients
Madalosso, 2009	Intervention – no physical manoeuver
Madalosso, 2010	Design – conference abstract
Martin-Du Pan, 2014	Design – narrative review
Melby, 2004	Design – narrative review
Morand, 2014	Population – blood donors
Morand, 2015	Population – blood donors
Morand, 2016	Population – blood donors
Müller, 2016	Language – Slovakian
Ntusi, 2015	Design – narrative review
Pancheva, 2006	Design – letter to the editor

Parry, 1999	Design – narrative review
Pauwels, 2012	Design – systematic review
Peterson, 2006	Design – letter to the editor
Philips, 2011	Design – narrative review
Podoleanu, 2006	Intervention – no physical manoeuver
Podoleanu, 2009	Intervention – no physical manoeuver
Protheroe, 2010	Intervention – no physical manoeuver
Santos, 2013	Intervention – no physical manoeuver
Sheldon, 2011	Design – guideline
Smit, 1997	Population – Orthostatic hypotension
Tanaka, 2014	Intervention – no physical manoeuver
Ten Harkel, 1994	Population – Orthostatic hypotension
Thijs, 2007	Population – Orthostatic hypotension
Tomaino, 2014	Design – conference abstract
Tutaj, 2006	Population – Orthostatic hypotension
Van Lieshout, 1992	Population – Orthostatic hypotension
Wieling, 1996	Language – Dutch
Wieling, 2004	Design – narrative review
