# Supplementary material for: Physical manoeuvers as a preventive intervention to manage vasovagal syncope: A systematic review
Source: PLoS One. 2019 Feb 28;14(2):e0212012. doi: 10.1371/journal.pone.0212012 (PMC6395036; doi:10.1371/journal.pone.0212012)
Supplement: S2 Table — (PDF) [file pone.0212012.s004.pdf]

**Author, year, Country**	**Study design**	**Population**	**Comparison**	**Outcomes**
Alizadeh, 2016, Iran	Experimental: Randomised controlled trial	*Number of participants*:<br>n=96 vasovagal syncope participants<br>- 68 males<br>- 28 females<br><br>Three arms:<br>Intervention1 n=34<br>Intervention2 n=37<br>Control n=25<br><br>*Mean age ±SD:*<br>42.12 ±15.11 years	**<u>Intervention 1:</u>**<br>Daily life advice: Hand grip manoeuvre + salt and water consumption<br><br>**<u>Intervention 2:</u>**<br>Daily life advice: Squatting + salt and water consumption<br><br>**<u>Control:</u>**<br>Daily life advice: Salt and water consumption	Follow-up:<br>Visit in out-patient clinic<br>Frequency: every month<br>Duration: 2 years<br><br>Outcomes:<br>- Compliance<br>- # people with syncope episodes<br>- # self-administered manoeuvers<br><br>[only # people with syncope episodes was extracted – other data not well reported; authors did not respond to e-mail]
Brignole, 2002, Italy	Experimental: Randomised cross-over trial	*Number of participants*:<br>n=19 vasovagal syncope participants<br>- 12 males<br>- 7 females<br><br>*Mean age ±SD:*<br>55 ±20 years<br><br>*Number of participants*:<br>n=32 healthy participants<br><br>[Data on healthy participants was not extracted]	Head-up tilt test 60° (duration not reported)<br>If no syncope occurred, 0.4mg nitro-glycerine spray was administered.<br><br>**<u>Intervention:</u>** Hand grip= 2 minutes at 50% of maximum voluntary contraction, starting at the onset of symptoms of impending syncope (as measured using the Martin Vigorimeter cuff manometer)<br><br>**<u>Control:</u>**<br>Placebo hand grip= 2 minutes no contraction, starting at the onset of	Outcomes, analysed at 120 seconds after start of the intervention or upon syncope occurrence:<br>- Systolic BP<br>- Diastolic BP<br>- Heart rate<br>- Syncope symptoms (nausea/headache) during counter-pressure phase and during recovery phase<br>- # people with syncope episodes during counter-pressure phase, during recovery phase, and total<br>- # people without syncope or syncope symptoms

			symptoms of impending syncope<br><br>Intervention and control were counterbalanced, with at least 1 hour between the two tilt tests.	
	Experimental: before after study		**<u>Intervention:</u>**<br>Daily life advice:<br>Arm-tensing manoeuver at maximum tolerated voluntary contraction	Follow-up:<br>Visit in out-patient clinic<br>Frequency: every 3 months<br>Mean ±SD duration: 9 ±3 months<br><br>Outcomes:<br>- # people with syncope episodes<br>- # impending syncope<br>- # self-administered manoeuvers<br>- Satisfaction questionnaire<br><br>[Only # people with syncope episodes was extracted – other data not well reported]
Croci, 2004, Italy	Experimental: before after study	*Number of participants*:<br>n=29 vasovagal syncope participants<br>- 16 males<br>- 13 females<br><br>*Mean age ±SD:*<br>49 ±22 years	**<u>Intervention:</u>**<br>Daily life advice:<br>Arm-tensing and/or hand grip manoeuver + education	Follow-up:<br>Visit in out-patient clinic<br>Frequency: every 3 months<br>Mean ±SD duration: 14 ±6 months<br><br>Outcomes:<br>- # people with syncope episodes<br>- # impending syncope<br>- # self-administered manoeuvers<br>- Satisfaction questionnaire

				[Only # people with syncope episodes was extracted]
Kim, 2005, Korea	Experimental: Non-randomised cross-over trial	*Number of participants*: n=27 syncope participants with positive head up tilt test (HUT)<br>- 14 males<br>- 13 females<br><br>[only 21 participants performed manoeuvres]<br><br>*Mean ± SD age:* 44.5±15.3 years<br><br>*Number of participants*: n=23 syncope participants with negative HUT<br><br>*Number of participants*: n=21 healthy participants<br><br>[Data from negative HUT and healthy controls were not extracted]	6 minute supine, followed by 20 minute head-up tilt test (HUT) 60°. If no syncope occurred, nitro-glycerine spray was administered and tilt continued for 16 minutes<br><br>**Intervention1:**<br>30 s hand grip, 5 min before and after HUT<br><br>**Intervention2:**<br>30 s leg-cross + muscle tension, 5 min before and after Hut<br><br>**Intervention3:**<br>30 s squat, 5 min before and after HUT<br><br>In patients with positive HUT, one week later: 30 s hand grip, leg crossing, and squat were performed, during a HUT upon induction of prodromal symptoms	Outcomes, time point at which these were analysed not clearly defined:<br>- Systolic BP<br>- Diastolic BP<br>- Heart rate<br>- # people with syncope episodes<br><br>[Only # people with syncope episodes was extracted]
Krediet, 2002, Netherlands	Experimental: Interrupted time-series	*Number of participants*: n=21 vasovagal syncope participants<br>- 11 males<br>- 10 females	5 minute supine, followed by 20 minute head-up tilt test 60°<br>If no syncope occurred, 0.4mg nitro-glycerine spray was administered and tilt continued for 15 minutes	Outcomes, analysed as average over the 4.5-5 min interval during rest, 2.5-3 min during head-up tilt, 2-1.5 min before the first episode of leg crossing and 30 seconds after blood pressure stabilization by the physical manoeuver:

<table>
<tr><td rowspan="2"></td><td rowspan="2"></td><td rowspan="2">Mean age (range): 41 (17-74) years</td><td><u>Intervention:</u>
Leg crossing + lower body tensing
[The effects of leg crossing + lower body tensing were compared to hand grip in a separate experiment – these data were not fit for data extraction]</td><td>- Systolic BP
- Diastolic BP
- Heart rate</td></tr>
<tr><td><u>Intervention:</u>
Daily life advice: Leg crossing + lower body tensing</td><td>Follow-up:
Telephone call checks
Frequency: not reported
Duration: 10 months

Outcomes:
- # syncope episodes
- # self-administered manoeuvers

[Only # syncope episodes was extracted]</td></tr>
<tr><td>Krediet, 2005, Netherlands</td><td>Experimental:
<u>Intervention 1 and 2:</u>
Non-randomised controlled trial

<u>Intervention 3 and 4:</u>
Interrupted time-series</td><td>Number of participants:
n=26 vasovagal syncope participants

[Some participants took part in more than 1 intervention]

Four arms:
Intervention1 n=12
- 5 males
- 7 females
Intervention2 n=9
- 5 males
- 4 females</td><td>5 minute supine, followed by 20 minute head-up tilt test 60°
If no syncope occurred, 0.4mg nitro-glycerine spray was administered and tilt continued for 15 minutes

<u>Intervention1:</u>
Leg crossing with muscle tensing (LCMT) + lower body muscle tensing (LBMT)

<u>Intervention2:</u>
Whole body muscle tensing (WBT) + LCMT</td><td>Outcomes, analysed as average over the 4.5-5 min interval during rest, 2.5-3 min during head-up tilt and 30 seconds after blood pressure stabilization by the physical manoeuver:
- Systolic BP
- Diastolic BP
- Mean arterial pressure
- Heart rate
- Stroke volume
- Cardiac output
- Total peripheral resistance</td></tr>
</table>

		Intervention3<br>n=14<br>- 5 males<br>- 9 females<br>Intervention4<br>n=9<br>- 2 males<br>- 7 females<br><br>*Median age (range):*<br>Intervention1:<br>45 (18-80) years<br><br>Intervention2:<br>37 (16-71) years<br><br>Intervention3:<br>27 (16-52) years<br><br>Intervention4:<br>28 (16-38) years	**Intervention3:**<br>Squatting (SQT)<br><br>**Intervention4:**<br>Sitting with head bent between the knees (HBK)	
Krediet, 2008, Netherlands	Experimental: Non-randomised cross-over trial	*Number of participants*:<br>n=18 vasovagal syncope participants<br>- 8 males<br>- 10 females<br><br>*Median age (range):*<br>37.5 (18-65) years	**No tilt-control:**<br>- 5 min supine rest<br>- 5 min free standing<br>- 1 min squat<br>- 1 sec rise<br>- 1 min stand<br><br>5 minute supine, followed by 20 minute head-up tilt test 60°<br>If no syncope occurred, 0.4mg nitro-glycerine spray was administered and tilt continued for 15 minutes.<br><br>When prodromal symptoms occur:<br>**Tilt control:**<br>- 1-5 min supine rest<br>- 1 min squat	Outcomes, analysed over the -20 to -5 interval during rest and at nadir BP during the physical manoeuvers:<br>- Systolic BP<br>- Diastolic BP<br>- Heart rate<br>- Stroke volume<br>- Cardiac output<br>- Total peripheral resistance<br>- Mean arterial pressure<br>- # people with syncope symptoms<br><br>[Heart rate and stroke volume were not extracted – not well reported]

			- 1 sec rise<br>- 1 min stand<br><br>**Intervention:**<br>- 1-5 min supine rest<br>- 1 min squat<br>- 1 sec rise<br>- 30-40 s lower body muscle tensing	
Romme, 2010, Netherlands	Experimental: Before after study	*Number of participants:* n=100 vasovagal syncope participants<br>- 34 males<br>- 66 females<br><br>*Mean age ± SD:* 38 ±14 years	**Intervention:**<br>Daily life advice: physical manoeuvers (leg crossing, lower body tension, squat, hand grip, arm tension) + Avoid syncope triggers + adequate fluid intake + salt intake +no excessive alcohol consumption + exercise regularly.	Follow-up:<br>Telephone call checks or outpatient visit + logbook<br>Frequency: every 3 months<br>Mean duration: 12 months<br><br>Outcomes:<br>- # people with syncope episodes<br>- # self-administered manoeuvers<br>- Quality of life<br><br>[Only # people with syncope episodes was extracted]
Tomaino, 2014, Italy	Experimental: Non-randomised controlled trial	*Number of participants*: n= 85 severe neurally mediated syncope participants, people with syncope due to a) bradycardia, b) slight/no rhythm variations, c) sinus tachycardia<br><br>Two arms:	**Intervention:**<br>Daily life advice: physical counter-pressure manoeuver therapy + lifestyle changes (avoiding situations that led to syncope)<br><br>Manoeuvers: leg crossing (as preventive measure) and lower body muscle tensing or hand grip and arm tensing (in case of	Follow-up:<br>Visits + logbook<br>Frequency: quarterly<br>Mean ±SD duration: 16 ±10 months<br><br>Outcomes:<br>- Time to first syncope recurrence<br>- Symptoms<br>- # people experiencing syncope<br><br>[Symptoms were not extracted – not well reported]

		Intervention n=40<br>- 20 males<br>- 20 females<br><br>Control n=45<br>- 14 males<br>- 31 females<br><br>*Mean age ±SD:*<br>Intervention:<br>62 ±13 years<br><br>Control:<br>66 ±12 years	occurrence of symptoms)<br><br>**Control:**<br>No intervention.	
Van Dijk, 2005, Netherlands	Experimental: Interrupted time-series	*Number of participants*:<br>n=88 vasovagal syncope participants<br>- 42 males<br>- 46 females<br><br>*Median age (range):*<br>38.5 (16-85) years	**Intervention:**<br>- 5 min supine rest<br>- 5 min standing<br>- 2 min legs crossed<br>- 1 min lower body muscle tensing	Outcomes, analysed over the 4.5-5 min interval after standing up and during the whole interval of physical manoeuvers, disregarding the first 10 seconds:<br>- Systolic BP<br>- Diastolic BP<br>- Heart rate<br>- Mean arterial pressure<br>- Pulse pressure<br>- Stroke volume<br>- Cardiac output<br>- Total peripheral resistance<br><br>[pulse pressure was not extracted]
Van Dijk, 2006, Netherlands	Experimental: Randomised controlled trial	*Number of participants*:<br>n=223 vasovagal syncope participants from 15 medical centres worldwide<br><br>[15 lost to follow-up]	**Intervention:**<br>Daily life advice: Conventional therapy* + physical counter-pressure manoeuvers – leg crossing, hand grip, arm tensing<br><br>**Control:**	Follow-up:<br>Visits<br>Frequency: Twice<br>Duration: 1 year<br><br>Telephone call checks<br>Frequency: 3 months<br>Duration (range): 6-18 months, mean IC: 14.4±5.4 months, CG: 14.1±5.1 months

		Intervention n=98<br>- 39 males<br>- 59 females<br><br>Control n=110<br>- 31 males<br>- 79 females<br><br>*Mean age ±SD:*<br>Intervention:<br>37.3 ±14.6 years<br><br>Control:<br>38.6 ±15.4 years	Daily life advice: Conventional therapy*<br><br>*Conventional therapy = advice to avoid triggers, lying down in case of symptoms and increasing fluid and salt intake + educational session explaining the mechanisms of vasovagal syncope	Outcomes:<br>- # syncope episodes<br>- # syncope recurrences<br>- # self-administered manoeuvers<br>- Time to first recurrence of syncope<br>- Logbook with registration of symptoms<br><br>[# self-administered manoeuvers and logbook data were not extracted]
