# Supplementary material for: Physical manoeuvers as a preventive intervention to manage vasovagal syncope: A systematic review
Source: PLoS One. 2019 Feb 28;14(2):e0212012. doi: 10.1371/journal.pone.0212012 (PMC6395036; doi:10.1371/journal.pone.0212012)
Supplement: S3 Table — (PDF) [file pone.0212012.s005.pdf]

Outcomes	Number of participants (studies)	Limitations in study design	Consistency	Indirectness	Imprecision	Publication bias	Quality of the evidence (GRADE)
All PM							
Syncope prevalence	563 (9 studies)	-1[a]	0	0	-1[c]	0	Low
PM shortening the hydrostatic column							
Head between knees							
SBP	9 (1 study)	-1[a]	0	0	-1[c]	0	Low
DBP	9 (1 study)	-1[a]	0	0	-1[c]	0	Low
MAP	9 (1 study)	-1[a]	0	0	-1[c]	0	Low
HR	9 (1 study)	-1[a]	0	0	-1[c]	0	Low
SV	9 (1 study)	-1[a]	0	0	-1[c]	0	Low
CO	9 (1 study)	-1[a]	0	0	-1[c]	0	Low
TPR	9 (1 study)	-1[a]	0	0	-1[c]	0	Low
PM using mechanical compression of the veins							
Hand grip							
Syncope prevalence	126 (3 studies)	-1[a]	0	0	-1[c]	0	Low
SBP	19 (1 study)	-1[a]	0	0	-1[c]	0	Low
DBP	19 (1 study)	-1[a]	0	0	-1[c]	0	Low
MAP	- (0 studies)	-	-	-	-	-	-
HR	19 (1 study)	-1[a]	0	0	-1[c]	0	Low
SV	-	-	-	-	-	-	-

	(0 studies)						
CO	-<br>(0 studies)	-	-	-	-	-	-
TPR	-<br>(0 studies)	-	-	-	-	-	-
Leg crossing							
SBP	88<br>(1 studies)	-1[a]	0	0	-1[c]	0	Low
DBP	88<br>(1 studies)	-1[a]	0	0	-1[c]	0	Low
MAP	88<br>(1 studies)	-1[a]	0	0	-1[c]	0	Low
HR	88<br>(1 studies)	-1[a]	0	0	-1[c]	0	Low
SV	88<br>(1 studies)	-1[a]	0	0	-1[c]	0	Low
CO	88<br>(1 studies)	-1[a]	0	0	-1[c]	0	Low
TPR	88<br>(1 studies)	-1[a]	0	0	-1[c]	0	Low
Squatting							
SBP	14<br>(1 studies)	-1[a]	0	0	-1[c]	0	Low
DBP	14<br>(1 studies)	-1[a]	0	0	-1[c]	0	Low
MAP	14<br>(1 studies)	-1[a]	0	0	-1[c]	0	Low
HR	14<br>(1 studies)	-1[a]	0	0	-1[c]	0	Low
SV	14<br>(1 studies)	-1[a]	0	0	-1[c]	0	Low
CO	14<br>(1 studies)	-1[a]	0	0	-1[c]	0	Low
TPR	14<br>(1 studies)	-1[a]	0	0	-1[c]	0	Low

Lower body muscle tension							
SBP	30 (2 studies)	-1[a]	0	0	-1[c]	0	Low
DBP	30 (2 studies)	-1[a]	0	0	-1[c]	0	Low
MAP	30 (2 studies)	-1[a]	0	0	-1[c]	0	Low
HR	30 (2 studies)	-1[a]	0	0	-1[c]	0	Low
SV	30 (2 studies)	-1[a]	0	0	-1[c]	0	Low
CO	30 (2 studies)	-1[a]	0	0	-1[c]	0	Low
TPR	30 (2 studies)	-1[a]	0	0	-1[c]	0	Low
Whole body muscle tension							
SBP	9 (1 study)	-1[a]	0	0	-1[c]	0	Low
DBP	9 (1 study)	-1[a]	0	0	-1[c]	0	Low
MAP	9 (1 study)	-1[a]	0	0	-1[c]	0	Low
HR	9 (1 study)	-1[a]	0	0	-1[c]	0	Low
SV	9 (1 study)	-1[a]	0	0	-1[c]	0	Low
CO	9 (1 study)	-1[a]	0	0	-1[c]	0	Low
TPR	9 (1 study)	-1[a]	0	0	-1[c]	0	Low
Leg crossing with muscle tension							
SBP	42 (3 studies)	-1[a]	0	0	-1[c]	0	Low
DBP	42 (3 studies)	-1[a]	0	0	-1[c]	0	Low

MAP	42 (3 studies)	-1[a]	0	0	-1[c]	0	Low
HR	42 (3 studies)	-1[a]	0	0	-1[c]	0	Low
SV	42 (3 studies)	-1[a]	0	0	-1[c]	0	Low
CO	42 (3 studies)	-1[a]	0	0	-1[c]	0	Low
TPR	42 (3 studies)	-1[a]	-1[b]	0	-1[c]	0	Very low

PM: physical manoeuver; SBP: systolic blood pressure; DBP: diastolic blood pressure; MAP: mean arterial pressure; HR: heart rate; SV: stroke volume; CO: cardiac output; TPR: total peripheral resistance.

[a] Limitations in study design – see Risk of bias Figures 2 and 3

[b] Conflicting results between trials

[c] Imprecision due to limited sample sizes, low number of events, lack of data, and/or a large variability of results
