# Supplementary material for: Physical manoeuvers as a preventive intervention to manage vasovagal syncope: A systematic review
Source: PLoS One. 2019 Feb 28;14(2):e0212012. doi: 10.1371/journal.pone.0212012 (PMC6395036; doi:10.1371/journal.pone.0212012)
Supplement: S4 Table — (PDF) [file pone.0212012.s006.pdf]

Outcome	Comparison/Risk factor	Effect Size	#studies, # participants	Reference
Primary outcome: Syncope prevalence				
Daily life interventions				
# people with syncope episodes	Daily life setting: Manoeuvers vs control	1 year post:<br>Not statistically significant<br>39/71 vs 8/25 §<br>RR: 1.72, 95% CI [0.93;3.16]<br>(p=0.08)*	1, 71 vs 25	Alizadeh, 2016
# people with syncope episodes	Daily life setting: Manoeuvers vs control	2 year post:<br>Not statistically significant<br>10/71 vs 3/25 §<br>RR: 1.17, 95% CI [0.35;3.92]<br>(p=0.79)*	1, 71 vs 25	Alizadeh, 2016
# people with syncope episodes	Daily life setting: Manoeuvers vs control	Not statistically significant:<br>15/40 vs 24/45 §<br>RR: 0.70, 95% CI [0.43;1.14]<br>(p=0.15)*	1, 40 vs 45	Tomaino, 2014
# people with syncope episodes	Daily life setting: Manoeuvers vs control	Statistically significant:<br>31/98 vs 56/110 §<br>RR: 0.62, 95%CI [0.44;0.88]<br>(p=0.007)*<br>*In favour of manoeuvers*	1, 98 vs 110	Van Dijk, 2006
# people with syncope episodes	Daily life setting: Hand grip vs control	1 year post:<br>Not statistically significant:<br>16/34 vs 8/25 §<br>RR: 1.47, 95%CI [0.75;2.88] ¥<br>(p=0.26)*	1, 34 vs 25	Alizadeh, 2016
# people with syncope episodes	Daily life setting: Hand grip vs control	2 year post:<br>Not statistically significant:<br>6/34 vs 3/25 §<br>RR: 1.47, 95%CI [0.41;5.32] ¥<br>(p=0.56)*	1, 34 vs 25	Alizadeh, 2016
# people with syncope episodes	Daily life setting: Hand grip vs squat	1 year post:<br>Not statistically significant:<br>16/34 vs 23/37 §<br>RR: 0.76, 95%CI [0.49;1.17] ¥<br>(p=0.21)*	1, 34 vs 37	Alizadeh, 2016
# people with syncope episodes	Daily life setting: Hand grip vs squat	2 year post:<br>Not statistically significant:<br>6/34 vs 4/37 §<br>RR: 1.63, 95%CI [0.50;5.29] ¥<br>(p=0.41)*	1, 34 vs 37	Alizadeh, 2016

# people with syncope episodes	Daily life setting:<br>Squat vs control	1 year post:<br>Statistically significant:<br>23/37 vs 8/25 §<br>RR: 1.94, 95%CI [1.04;3.63]<br>(p=0.037)*<br>*In favour of control*	1, 37 vs 25	Alizadeh, 2016
# people with syncope episodes	Daily life setting:<br>Squat vs control	2 year post:<br>Not statistically significant:<br>4/37 vs 3/25 §<br>RR: 0.90, 95%CI [0.22;3.68] ¥<br>(p=0.88)*	1, 37 vs 25	Alizadeh, 2016
# people with syncope episodes	Daily life setting:<br>Post to pre<br>Manoeuvers	Statistically significant:<br>49/100 vs 100/100 §<br>RR: 0.49, 95% CI [0.40;0.60]<br>(p<0.00001)*<br>*In favour of manoeuvers*	1, 100<br>(within subject)	Romme, 2010
# people with syncope episodes	Daily life setting:<br>Post to pre<br>Arm tensing or hand grip	Statistically significant:<br>5/19 vs 19/19 §<br>RR: 0.28, 95% CI [0.14;0.57]<br>(p=0.0005)*<br>*In favour of arm tensing/hand grip*	1, 19<br>(within subject)	Croci, 2004
# people with syncope episodes	Daily life setting:<br>Post to pre<br>Arm tensing	Statistically significant:<br>1/11 vs 11/11 §<br>RR: 0.13, 95% CI [0.03;0.57]<br>(p=0.007)*<br>*In favour of arm tensing*	1, 11<br>(within subject)	Brignole, 2002
# people with syncope episodes	Daily life setting:<br>Post to pre<br>Leg crossing with muscle tension	Statistically significant:<br>4/13 vs 13/13 §<br>RR: 0.33, 95% CI [0.14;0.69]<br>(p=0.008)*<br>*In favour of LCMT*	1, 13<br>(within subject)	Krediet, 2002
Median yearly number syncope episodes/patient	Daily life setting:<br>Manoeuvers vs control	Statistically significant:<br>0.0 (0.0-0.7) vs 0.6 (0.0-1.3)<br>Median difference: 0.6*£†<br>(p=0.004)<br>*In favour of manoeuvers*	1, 98 vs 110 §	Van Dijk, 2006
# people with pre-syncopal episodes	Daily life setting:<br>Manoeuvers vs control	Not statistically significant:<br>81/98 vs 81/110 §<br>RR: 1.12, 95%CI [0.38;0.92]*<br>(p=0.118)	1, 98 vs 110 §	Van Dijk, 2006
Time to first recurrence	Daily life setting:<br>Manoeuvers vs control	Not statistically significant:<br>4.8±4.5 vs 6.6±5.9<br>MD: 1.8*£†<br>(p=0.106)	1, 98 vs 110 §	Van Dijk, 2006

Syncope-free survival	Daily life setting: Manoeuvers vs control	<u>Statistically significant:</u> HR: 0.59, 95%CI [0.38;0.92] (p=0.018) *In favour of manoeuvers*	1, 98 vs 110 §	Van Dijk, 2006
Mean monthly number of syncope episodes/patient	Daily life setting: Post to pre Leg crossing with muscle tension	0.02±0.06 vs 0.53±0.76 MD: -0.51 *£† [p not reported]	1, 13 vs 19 (within subject)	Krediet, 2002
Laboratory trials				
# people with syncope episodes	Laboratory setting: Post to pre Manoeuvers	<u>Statistically significant:</u> 8/13 vs 13/13 § RR: 0.63, 95% CI [0.39;0.97] (p=0.07)* *In favour of manoeuvers*	1, 13 (within subject)	Kim, 2005
# people with syncope episodes	Laboratory setting: Post to pre Hand grip	Not statistically significant: 12/13 vs 13/13 § RR: 0.93, 95%CI [0.74;1.14]* ¥ (p=1.0)	1, 13 (within subject)	Kim, 2005
# people with syncope episodes	Laboratory setting: Post to pre Squat	<u>Statistically significant:</u> 6/13 vs 13/13 § RR: 0.48, 95%CI [0.27;0.84]* (p=0.01) *In favour of squat*	1, 13 (within subject)	Kim, 2005
# people with syncope episodes	Laboratory setting: Post to pre Leg crossing with muscle tension	<u>Statistically significant:</u> 6/13 vs 13/13 § RR: 0.48, 95%CI [0.27;0.84]* (p=0.01) *In favour of LCMT*	1, 13 (within subject)	Kim, 2005
# people with syncope episodes	Laboratory setting: Hand grip vs control	<u>Statistically significant:</u> 4/19 vs 11/19 § RR: 0.36, 95%CI [0.17;0.8]$* (p=0.02) *In favour of hand grip*	1, 19 § (within subject)	Brignole, 2002
# people with pre-syncope symptoms	Laboratory setting: Post to pre Lower body tension	<u>Statistically significant:</u> 2/18 vs 13/18 § RR: 0.15, 95%CI [0.05;0.52]$* (p<0.01) *In favour of LBMT*	1, 18 (within subject)	Krediet, 2008
# people with syncope episodes (End of counter-pressure phase)	Laboratory setting: Hand grip vs control	<u>Statistically significant:</u> 1/19 vs 9/19 § RR: 0.11, 95%CI [0.02;0.95]$* (p=0.01) *In favour of hand grip*	1, 19 § (within subject)	Brignole, 2002

# people with syncope episodes (Recovery phase)	Laboratory setting: Hand grip vs control	Not statistically significant: 3/18 vs 2/10 § RR: 0.83, 95%CI [0.17;4.18]* ¥ (p=0.82)	1, 19 § (within subject)	Brignole, 2002
Syncope symptoms (End of counter-pressure phase)	Laboratory setting: Hand grip vs control	Not statistically significant: 0/19 vs 2/19 § RR: 0.2*£† (p>0.05)	1, 19 § (within subject)	Brignole, 2002
Syncope symptoms (Recovery phase)	Laboratory setting: Hand grip vs control	Not statistically significant: 0/18 vs 2/10 § RR: 0.12, 95%CI [0.01;2.2]* ¥ (p=0.15)	1, 19 § (within subject)	Brignole, 2002
# people without syncope or syncope symptoms (End of counter-pressure phase)	Laboratory setting: Hand grip vs control	<u>Statistically significant:</u> 12/19 vs 2/19 § RR: 6*£† (p=0.02) *In favour of hand grip*	1, 19 § (within subject)	Brignole, 2002
# people without syncope or syncope symptoms (Recovery phase)	Laboratory setting: Hand grip vs control	Not statistically significant: 9/19 vs 2/10 § RR: 2.37, 95%CI [0.63;8.93]* ¥ (p=0.2)	1, 19 § (within subject)	Brignole, 2002
Secondary outcomes				
1. PM shortening the hydrostatic column between the heart and the brain				
Systolic BP (mmHg)	Laboratory setting: Post to pre Head between knees	<u>Statistically significant:</u> 115±16 vs 69±8 MD: 46 *£† (p<0.05) *In favour of HBK*	1, 9 § (within subject)	Krediet, 2005
Diastolic BP (mmHg)	Laboratory setting: Post to pre Head between knees	<u>Statistically significant:</u> 70±16 vs 48±7 MD: 22 *£† (p<0.05) *In favour of HBK*	1, 9 § (within subject)	Krediet, 2005
Mean arterial pressure (mmHg)	Laboratory setting: Post to pre Head between knees	<u>Statistically significant:</u> 82±15 vs 53±8 MD: 29 *£† (p<0.05) *In favour of HBK*	1, 9 § (within subject)	Krediet, 2005
Heart rate (bpm)	Laboratory setting: Post to pre Head between knees	<u>Statistically significant:</u> 78±12 vs 99±14 MD: -21 *£† (p<0.05) *In favour of HBK*	1, 9 § (within subject)	Krediet, 2005
Stroke volume (%)	Laboratory setting: Post to pre Head between knees	<u>Statistically significant:</u> 98±30 vs 50±12 MD: 48 *£† (p<0.05) *In favour of HBK*	1, 9 § (within subject)	Krediet, 2005

Cardiac output (%)	Laboratory setting:<br>Post to pre<br>Head between knees	Statistically significant:<br>84±15 vs 57±16<br>MD: 27 *£†<br>(p<0.05)<br>*In favour of HBK*	1, 9 §<br>(within subject)	Krediet, 2005
Total peripheral resistance (%)	Laboratory setting:<br>Post to pre<br>Head between knees	Not statistically significant:<br>117±38 vs 127±51<br>MD: -10 *£†<br>(p>0.05)	1, 9 §<br>(within subject)	Krediet, 2005
2. PM using mechanical compression of the veins				
3.1. HAND GRIP				
Systolic BP (mmHg)	Laboratory setting:<br>Hand grip vs control	Statistically significant:<br>105±38 vs 73±21<br>MD: 32, 95%CI [9.31;54.96]$*<br>(p=0.008)<br>*In favour of hand grip*	1, 19 §<br>(within subject)	Brignole, 2002
Diastolic BP (mmHg)	Laboratory setting:<br>Hand grip vs control	Statistically significant:<br>71±24 vs 51±20<br>MD: 20, 95%CI [7.16;32.84]$*<br>(p=0.004)<br>*In favour of hand grip*	1, 19 §<br>(within subject)	Brignole, 2002
Heart rate (bpm)	Laboratory setting:<br>Hand grip vs control	Not statistically significant:<br>85±25 vs 77±20<br>MD: 8*£†<br>(p>0.05)	1, 19 §<br>(within subject)	Brignole, 2002
2.2. LEG CROSSING				
Systolic BP (mmHg)	Laboratory setting:<br>Post to pre<br>Leg crossing	Statistically significant:<br>130.9±16.9 vs 125.3±16.1<br>MD: 5.6*£†<br>(p<0.001)<br>*In favour of leg crossing*	1, 88 §<br>(within subject)	Van Dijk, 2005
Diastolic BP (mmHg)	Laboratory setting:<br>Post to pre<br>Leg crossing	Statistically significant:<br>75.0±10.7 vs 73.8±10.3<br>MD: 1.2 *£†<br>(p<0.01)<br>*In favour of leg crossing*	1, 88 §<br>(within subject)	Van Dijk, 2005
Mean arterial pressure (mmHg)	Laboratory setting:<br>Post to pre<br>Leg crossing	Statistically significant:<br>92.9±11.6 vs 89.9±11.1<br>MD: 3.0 *£†<br>(p<0.001)<br>*Increase with leg crossing*	1, 88 §<br>(within subject)	Van Dijk, 2005
Heart rate (bpm)	Laboratory setting:<br>Post to pre<br>Leg crossing	Not statistically significant:<br>82.2±14.9 vs 82.8±15.3<br>MD: -0.6 *£†<br>(p>0.05)	1, 88 §<br>(within subject)	Van Dijk, 2005
Stroke volume change (%)	Laboratory setting:<br>Post to pre<br>Leg crossing	Statistically significant:<br>10.7% £†<br>(p<0.001)<br>*In favour of leg crossing*	1, 88 §<br>(within subject)	Van Dijk, 2005

Cardiac output change (%)	Laboratory setting:<br>Post to pre<br>Leg crossing	<u>Statistically significant:</u><br>9.5% £†<br>(p<0.001)<br>*In favour of leg crossing*	1, 88 §<br>(within subject)	Van Dijk, 2005
Total peripheral resistance change (%)	Laboratory setting:<br>Post to pre<br>Leg crossing	<u>Statistically significant:</u><br>-5.1% £†<br>(p<0.001)<br>*In favour of control*	1, 88 §<br>(within subject)	Van Dijk, 2005
2.3. SQUAT				
Systolic BP (mmHg)	Laboratory setting:<br>Post to pre<br>Squat	<u>Statistically significant:</u><br>122±15 vs 76±13<br>MD: 46 *£†<br>(p<0.05)<br>*In favour of squat*	1, 14 §<br>(within subject)	Krediet, 2005
Diastolic BP (mmHg)	Laboratory setting:<br>Post to pre<br>Squat	<u>Statistically significant:</u><br>74±10 vs 50±10<br>MD: 24 *£†<br>(p<0.05)<br>*In favour of squat*	1, 14 §<br>(within subject)	Krediet, 2005
Mean arterial pressure (mmHg)	Laboratory setting:<br>Post to pre<br>Squat	<u>Statistically significant:</u><br>88±11 vs 57±12<br>MD: 31 *£†<br>(p<0.05)<br>*In favour of squat*	1, 14 §<br>(within subject)	Krediet, 2005
Heart rate (bpm)	Laboratory setting:<br>Post to pre<br>Squat	Not statistically significant:<br>80±8 vs 90±21<br>MD: -10 *£†<br>(p>0.05)	1, 14 §<br>(within subject)	Krediet, 2005
Stroke volume (%)	Laboratory setting:<br>Post to pre<br>Squat	<u>Statistically significant:</u><br>115±15 vs 65±19<br>MD: 50 *£†<br>(p<0.05)<br>*In favour of squat*	1, 14 §<br>(within subject)	Krediet, 2005
Cardiac output (%)	Laboratory setting:<br>Post to pre<br>Squat	<u>Statistically significant:</u><br>104±15 vs 66±23<br>MD: 38 *£†<br>(p<0.05)<br>*In favour of squat*	1, 14 §<br>(within subject)	Krediet, 2005
Total peripheral resistance (%)	Laboratory setting:<br>Post to pre<br>Squat	Not statistically significant:<br>99±16 vs 107±33<br>MD: -8 *£†<br>(p>0.05)	1, 14 §<br>(within subject)	Krediet, 2005
2.4. LOWER BODY MUSCLE TENSION				
Systolic BP (mmHg)	Laboratory setting:<br>Lower body tension vs control	103±3 vs 84±5<br>MD: 19 *£†<br>[p not reported]	1, 18 §<br>(within subject)	Krediet, 2008
Systolic BP (mmHg)	Laboratory setting:<br>Post to pre<br>Lower body tension	<u>Statistically significant:</u><br>104±18 vs 77±8<br>MD: 27 *£†<br>(p<0.05)<br>*In favour of LBMT*	1, 12 §<br>(within subject)	Krediet, 2005

Systolic BP (mmHg)	Laboratory setting: Lower body tension vs leg crossing with muscle tension	Statistically significant: 104±18 vs 120±13 MD: -16 *£† ($p<0.05$) *In favour of LCMT*	1, 12 § (within subject)	Krediet, 2005
Diastolic BP (mmHg)	Laboratory setting: Lower body tension vs control	65±3 vs 55±3 MD: 10 *£† [p not reported]	1, 18 § (within subject)	Krediet, 2008
Diastolic BP (mmHg)	Laboratory setting: Post to pre Lower body tension	Statistically significant: 69±12 vs 54±8 MD: 15 *£† ($p<0.05$) *In favour of LBMT*	1, 12 § (within subject)	Krediet, 2005
Diastolic BP (mmHg)	Laboratory setting: Lower body tension vs leg crossing with muscle tension	Not statistically significant: 69±12 vs 76±11 MD: -7 *£† ($p>0.05$)	1, 12 § (within subject)	Krediet, 2005
Mean arterial pressure (mmHg)	Laboratory setting: Lower body tension vs control	Statistically significant: 76±3 vs 64±4 MD: 12 *£† ($p<0.001$) *In favour of LBMT*	1, 18 § (within subject)	Krediet, 2008
Mean arterial pressure (mmHg)	Laboratory setting: Post to pre Lower body tension	Statistically significant: 79±13 vs 61±7 MD: 18 *£† ($p<0.05$) *In favour of LBMT*	1, 12 § (within subject)	Krediet, 2005
Mean arterial pressure (mmHg)	Laboratory setting: Lower body tension vs leg crossing with muscle tension	Statistically significant: 79±13 vs 89±11 MD: -10 *£† ($p<0.05$) *In favour of LCMT*	1, 12 § (within subject)	Krediet, 2005
Heart rate (bpm)	Laboratory setting: Post to pre Lower body tension	Statistically significant: 103±20 vs 96±28 MD: 7 *£† ($p<0.05$) *In favour of LBMT*	1, 12 § (within subject)	Krediet, 2005
Heart rate (bpm)	Laboratory setting: Lower body tension vs leg crossing with muscle tension	Not statistically significant: 103±20 vs 97±16 MD: 6 *£† ($p>0.05$)	1, 12 § (within subject)	Krediet, 2005
Stroke volume (%)	Laboratory setting: Post to pre Lower body tension	Statistically significant: 83±25 vs 66±19 MD: 17 *£† ($p<0.05$) *In favour of LBMT*	1, 12 § (within subject)	Krediet, 2005
Stroke volume (%)	Laboratory setting: Lower body tension vs leg crossing with muscle tension	Statistically significant: 83±25 vs 100±24 MD: -17 *£† ($p<0.05$) *In favour of LCMT*	1, 12 § (within subject)	Krediet, 2005

Cardiac output (%)	Laboratory setting: Lower body tension vs control	Statistically significant: 85±6 vs 73±5 MD: 12 *£† (p=.0009) *In favour of LBMT*	1, 18 § (within subject)	Krediet, 2008
Cardiac output (%)	Laboratory setting: Post to pre Lower body tension	Statistically significant: 95±21 vs 69±12 MD: 26 *£† (p<0.05) *In favour of LBMT*	1, 12 § (within subject)	Krediet, 2005
Cardiac output (%)	Laboratory setting: Lower body tension vs leg crossing with muscle tension	Statistically significant: 95±21 vs 110±22 MD: -15 *£† (p<0.05) *In favour of LCMT*	1, 12 § (within subject)	Krediet, 2005
Total peripheral resistance (%)	Laboratory setting: Lower body tension vs control	Not statistically significant 101±7 vs 99±5 MD: 2 *£† (p=0.34)	1, 18 § (within subject)	Krediet, 2008
Total peripheral resistance (%)	Laboratory setting: Post to pre Lower body tension	Not statistically significant: 99±20 vs 104±22 MD: -5 *£† (p>0.05)	1, 12 § (within subject)	Krediet, 2005
Total peripheral resistance (%)	Laboratory setting: Lower body tension vs leg crossing with muscle tension	Not statistically significant: 99±20 vs 96±21 MD: 3 *£† (p>0.05)	1, 12 § (within subject)	Krediet, 2005
2.5. WHOLE BODY MUSCLE TENSION				
Systolic BP (mmHg)	Laboratory setting: Whole body tension vs leg crossing with muscle tension	Statistically significant: 115±23 vs 123±19 MD: -8 *£† (p<0.05) *In favour of LCMT*	1, 9 § (within subject)	Krediet, 2005
Systolic BP (mmHg)	Laboratory setting: Post to pre Whole body tension	Statistically significant: 115±23 vs 73±6 MD: 42 *£† (p<0.05) *In favour of WBT*	1, 9 § (within subject)	Krediet, 2005
Diastolic BP (mmHg)	Laboratory setting: Whole body tension vs leg crossing with muscle tension	Not statistically significant: 73±16 vs 76±14 MD: -3 *£† (p>0.05)	1, 9 § (within subject)	Krediet, 2005
Diastolic BP (mmHg)	Laboratory setting: Post to pre Whole body tension	Statistically significant: 73±16 vs 51±8 MD: 22 *£† (p<0.05) *In favour of WBT*	1, 9 § (within subject)	Krediet, 2005
Mean arterial pressure (mmHg)	Laboratory setting: Whole body tension vs leg crossing with muscle tension	Statistically significant: 83±16 vs 88±14 MD: -5 *£† (p<0.05) *In favour of LCMT*	1, 9 § (within subject)	Krediet, 2005

Mean arterial pressure (mmHg)	Laboratory setting:<br>Post to pre<br>Whole body tension	<u>Statistically significant:</u><br>83±16 vs 56±7<br>MD: 27 *£†<br>(p<0.05)<br>*In favour of WBT*	1, 9 §<br>(within subject)	Krediet, 2005
Heart rate (bpm)	Laboratory setting:<br>Post to pre<br>Whole body tension	<u>Statistically significant:</u><br>110±21 vs 94±27<br>MD: 16 *£†<br>(p<0.05)<br>*In favour of WBT*	1, 9 §<br>(within subject)	Krediet, 2005
Heart rate (bpm)	Laboratory setting:<br>Whole body tension vs leg crossing with muscle tension	Not statistically significant:<br>110±21 vs 107±22<br>MD: 3 *£†<br>(p>0.05)	1, 9 §<br>(within subject)	Krediet, 2005
Stroke volume (%)	Laboratory setting:<br>Post to pre<br>Whole body tension	<u>Statistically significant:</u><br>80±23 vs 53±16<br>MD: 27 *£†<br>(p<0.05)<br>*In favour of WBT*	1, 9 §<br>(within subject)	Krediet, 2005
Stroke volume (%)	Laboratory setting:<br>Whole body tension vs leg crossing with muscle tension	Not statistically significant:<br>80±23 vs 90±28<br>MD: -10 *£†<br>(p>0.05)	1, 9 §<br>(within subject)	Krediet, 2005
Cardiac output (%)	Laboratory setting:<br>Post to pre<br>Whole body tension	<u>Statistically significant:</u><br>94±21 vs 54±12<br>MD: 40 *£†<br>(p<0.05)<br>*In favour of WBT*	1, 9 §<br>(within subject)	Krediet, 2005
Cardiac output (%)	Laboratory setting:<br>Whole body tension vs leg crossing with muscle tension	Not statistically significant:<br>94±21 vs 104±23<br>MD: -10 *£†<br>(p>0.05)	1, 9 §<br>(within subject)	Krediet, 2005
Total peripheral resistance (%)	Laboratory setting:<br>Post to pre<br>Whole body tension	Not statistically significant:<br>115±26 vs 137±38<br>MD: -22 *£†<br>(p>0.05)	1, 9 §<br>(within subject)	Krediet, 2005
Total peripheral resistance (%)	Laboratory setting:<br>Whole body tension vs leg crossing with muscle tension	Not statistically significant:<br>115±26 vs 112±34<br>MD: 3 *£†<br>(p>0.05)	1, 9 §<br>(within subject)	Krediet, 2005
2.6. LEG CROSSING WITH MUSCLE TENSION				
Systolic BP (mmHg)	Laboratory setting:<br>Leg crossing with muscle tension vs lower body tension	<u>Statistically significant:</u><br>120±13 vs 104±18<br>MD: 16 *£†<br>(p<0.05)<br>*In favour of LCMT*	1, 12 §<br>(within subject)	Krediet, 2005
Systolic BP (mmHg)	Laboratory setting:<br>Leg crossing with muscle tension vs whole body tension	<u>Statistically significant:</u><br>123±19 vs 115±23<br>MD: 8 *£†<br>(p<0.05)<br>*In favour of LCMT*	1, 9 §<br>(within subject)	Krediet, 2005

Systolic BP (mmHg)	Laboratory setting: Post to pre Leg crossing with muscle tension	Statistically significant: 106±16 vs 65±13 MD: 41 *£† ($p<0.001$) *In favour of LCMT*	1, 21 § (within subject)	Krediet, 2002
Systolic BP (mmHg)	Laboratory setting: Post to pre Leg crossing with muscle tension	Statistically significant: 120±13 vs 75±14 MD= 45 *£† ($p<0.05$) *In favour of LCMT*	1, 12 § (within subject)	Krediet, 2005
Systolic BP (mmHg)	Laboratory setting: Post to pre Leg crossing with muscle tension	Statistically significant: 123±19 vs 70±10 MD: 53 *£† ($p<0.05$) *In favour of LCMT*	1, 9 § (within subject)	Krediet, 2005
Systolic BP (mmHg)	Laboratory setting: Post to pre Leg crossing with muscle tension	Statistically significant: 142.2±18.1 vs 126.1±15.9 MD: 16.1 *£† ($p<0.001$) *In favour of LCMT*	1, 54 § (within subject)	Van Dijk, 2005
Diastolic BP (mmHg)	Laboratory setting: Leg crossing with muscle tension vs lower body tension	Not statistically significant: 76±11 vs 69±12 MD: 7 *£† ($p>0.05$)	1, 12 § (within subject)	Krediet, 2005
Diastolic BP (mmHg)	Laboratory setting: Leg crossing with muscle tension vs whole body tension	Not statistically significant: 76±14 vs 73±16 MD: 3 *£† ($p>0.05$)	1, 9 § (within subject)	Krediet, 2005
Diastolic BP (mmHg)	Laboratory setting: Post to pre Leg crossing with muscle tension	Statistically significant: 76±11 vs 53±11 MD: 23 *£† ($p<0.05$) *In favour of LCMT*	1, 12 § (within subject)	Krediet, 2005
Diastolic BP (mmHg)	Laboratory setting: Post to pre Leg crossing with muscle tension	Statistically significant: 76±14 vs 50±10 MD: 26 *£† ($p<0.05$) *In favour of LCMT*	1, 9 § (within subject)	Krediet, 2005
Diastolic BP (mmHg)	Laboratory setting: Post to pre Leg crossing with muscle tension	Statistically significant: 65±10 vs 43±9 MD: 22 *£† ($p<0.001$) *In favour of LCMT*	1, 21 § (within subject)	Krediet, 2002
Diastolic BP (mmHg)	Laboratory setting: Post to pre Leg crossing with muscle tension	Statistically significant: 81.8±11.4 vs 74.9±10.6 MD: 6.9 *£† ($p<0.001$) *In favour of LCMT*	1, 54 § (within subject)	Van Dijk, 2005
Mean arterial pressure (mmHg)	Laboratory setting: Leg crossing with muscle tension vs whole body tension	Statistically significant: 88±14 vs 83±16 MD: 5 *£† ($p<0.05$)	1, 9 § (within subject)	Krediet, 2005

		In favour of LCMT		
Mean arterial pressure (mmHg)	Laboratory setting: Leg crossing with muscle tension vs lower body tension	<u>Statistically significant:</u> 89±11 vs 79±13 MD: 10 *£† ($p<0.05$) *In favour of LCMT*	1, 12 § (within subject)	Krediet, 2005
Mean arterial pressure (mmHg)	Laboratory setting: Post to pre Leg crossing with muscle tension	<u>Statistically significant:</u> 89±11 vs 60±12 MD: 29 *£† ($p<0.05$) *Increase with LCMT*	1, 12 § (within subject)	Krediet, 2005
Mean arterial pressure (mmHg)	Laboratory setting: Post to pre Leg crossing with muscle tension	<u>Statistically significant:</u> 88±14 vs 55±10 MD: 33 *£† ($p<0.05$) *Increase with LCMT*	1, 9 § (within subject)	Krediet, 2005
Mean arterial pressure (mmHg)	Laboratory setting: Post to pre Leg crossing with muscle tension	<u>Statistically significant:</u> 101.1±12.6 vs 90.8±11.4 MD: 10.3 *£† ($p<0.001$) *In favour of LCMT*	1, 54 § (within subject)	Van Dijk, 2005
Heart rate (bpm)	Laboratory setting: Leg crossing with muscle tension vs lower body tension	Not statistically significant: 97±16 vs 103±20 MD: -6 *£† ($p>0.05$)	1, 12 § (within subject)	Krediet, 2005
Heart rate (bpm)	Laboratory setting: Leg crossing with muscle tension vs whole body tension	Not statistically significant: 107±22 vs 110±21 MD: -3 *£† ($p>0.05$)	1, 9 § (within subject)	Krediet, 2005
Heart rate (bpm)	Laboratory setting: Post to pre Leg crossing with muscle tension	<u>Statistically significant:</u> 97±16 vs 93±23 MD: 4 *£† ($p<0.05$) *In favour of LCMT*	1, 12 § (within subject)	Krediet, 2005
Heart rate (bpm)	Laboratory setting: Post to pre Leg crossing with muscle tension	<u>Statistically significant:</u> 107±22 vs 99±23 MD: 8 *£† ($p<0.05$) *In favour of LCMT*	1, 9 § (within subject)	Krediet, 2005
Heart rate (bpm)	Laboratory setting: Post to pre Leg crossing with muscle tension	<u>Statistically significant:</u> 82±15 vs 73±22 MD: 9 *£† ($p<0.01$) *In favour of LCMT*	1, 21 § (within subject)	Krediet, 2002
Heart rate (bpm)	Laboratory setting: Post to pre Leg crossing with muscle tension	<u>Statistically significant:</u> 90.0±16.0 vs 86.3±15.7 MD: 3.7 *£† ($p<0.01$) *In favour of LCMT*	1, 54 § (within subject)	Van Dijk, 2005
Stroke volume (%)	Laboratory setting:	Not statistically significant: 90±28 vs 80±23 MD: 10 *£†	1, 9 § (within subject)	Krediet, 2005

	Leg crossing with muscle tension vs whole body tension	($p>0.05$)		
Stroke volume (%)	Laboratory setting: Leg crossing with muscle tension vs lower body tension	<u>Statistically significant:</u><br>100±24 vs 83±25<br>MD: 17 *£†<br>($p<0.05$)<br>*In favour of LCMT*	1, 12 §<br>(within subject)	Krediet, 2005
Stroke volume (%)	Laboratory setting: Post to pre<br>Leg crossing with muscle tension	<u>Statistically significant:</u><br>100±24 vs 64±24<br>MD: 36 *£†<br>($p<0.05$)<br>*In favour of LCMT*	1, 12 §<br>(within subject)	Krediet, 2005
Stroke volume (%)	Laboratory setting: Post to pre<br>Leg crossing with muscle tension	<u>Statistically significant:</u><br>90±28 vs 55±19<br>MD: 35 *£†<br>($p<0.05$)<br>*In favour of LCMT*	1, 9 §<br>(within subject)	Krediet, 2005
Stroke volume change (%)	Laboratory setting: Post to pre<br>Leg crossing with muscle tension	<u>Statistically significant:</u><br>14.0% £†<br>($p<0.001$)<br>*In favour of LCMT*	1, 54 §<br>(within subject)	Van Dijk, 2005
Cardiac output (%)	Laboratory setting: Leg crossing with muscle tension vs whole body tension	Not statistically significant:<br>104±23 vs 94±21<br>MD: 10 *£†<br>($p>0.05$)	1, 9 §<br>(within subject)	Krediet, 2005
Cardiac output (%)	Laboratory setting: Leg crossing with muscle tension vs lower body tension	<u>Statistically significant:</u><br>110±22 vs 95±21<br>MD: 15 *£†<br>($p<0.05$)<br>*In favour of LCMT*	1, 12 §<br>(within subject)	Krediet, 2005
Cardiac output (%)	Laboratory setting: Post to pre<br>Leg crossing with muscle tension	<u>Statistically significant:</u><br>110±22 vs 65±17<br>MD: 45 *£†<br>($p<0.05$)<br>*In favour of LCMT*	1, 12 §<br>(within subject)	Krediet, 2005
Cardiac output (%)	Laboratory setting: Post to pre<br>Leg crossing with muscle tension	<u>Statistically significant:</u><br>104±23 vs 59±16<br>MD: 45 *£†<br>($p<0.05$)<br>*In favour of LCMT*	1, 9 §<br>(within subject)	Krediet, 2005
Cardiac output change (%)	Laboratory setting: Post to pre<br>Leg crossing with muscle tension	<u>Statistically significant:</u><br>19.6% £†<br>($p<0.001$)<br>*In favour of LCMT*	1, 54 §<br>(within subject)	Van Dijk, 2005
Total peripheral resistance (%)	Laboratory setting: Leg crossing with muscle tension vs whole body tension	Not statistically significant:<br>112±34 vs 115±26<br>MD: -3 *£†<br>($p>0.05$)	1, 9 §<br>(within subject)	Krediet, 2005
Total peripheral resistance (%)	Laboratory setting:	Not statistically significant:<br>96±21 vs 99±20<br>MD: -3 *£†	1, 12 §<br>(within subject)	Krediet, 2005

	Leg crossing with muscle tension vs lower body tension	(p>0.05)		
Total peripheral resistance (%)	Laboratory setting: Post to pre Leg crossing with muscle tension	Not statistically significant: 96±21 vs 62±30 MD: 34 *£† (p>0.05)	1, 12 § (within subject)	Krediet, 2005
Total peripheral resistance (%)	Laboratory setting: Post to pre Leg crossing with muscle tension	Not statistically significant: 112±34 vs 132±47 MD: -20 *£† (p>0.05)	1, 9 § (within subject)	Krediet, 2005
Total peripheral resistance change (%)	Laboratory setting: Post to pre Leg crossing with muscle tension	Statistically significant: -5.2% £† (p<0.01) *In favour of control*	1, 54 § (within subject)	Van Dijk, 2005

§: Imprecision (low sample size/low number of events)

£: Paired data, 95% CI could not be calculated

££: No raw data available, no summary estimated or 95% CI could be calculated

¥: Imprecision (wide confidence intervals)

†: Imprecision (lack of data)

*: Calculations were done using Review Manager 5.3

**: Calculations were done using R
